# Arithmetic fundamental lemma for the spherical Hecke algebra

**DOI:** 10.1007/s00229-024-01572-0

**Published:** 2024-06-20

**Authors:** Chao Li, Michael Rapoport, Wei Zhang

**Affiliations:** 1 Department of Mathematics, Columbia University, 2990 Broadway, New York, NY 10027 USA; 2 Mathematisches Institut der Universität Bonn, Endenicher Allee 60, 53115 Bonn, Germany; 3 Department of Mathematics, Massachusetts Institute of Technology, 77 Massachusetts Avenue, Cambridge, MA 02139 USA

**Keywords:** 11G18, 11F70, 14C25

## Abstract

We define Hecke correspondences and Hecke operators on unitary RZ spaces and study their basic geometric properties, including a commutativity conjecture on Hecke operators. Then we formulate the arithmetic fundamental lemma conjecture for the spherical Hecke algebra. We also formulate a conjecture on the abundance of spherical Hecke functions with identically vanishing first derivative of orbital integrals. We prove these conjectures for the case $\textrm{U} (1)\times \textrm{U} (2)$.

## Introduction

In the relative trace formula approach of Jacquet and Rallis to the Gan–Gross–Prasad conjecture, the Jacquet-Rallis fundamental lemma (FL) conjecture plays a key role [15]. It states an identity of the following form. Let *p* be an odd prime number. Let $F_0$ be a finite extension of $\mathbb {Q} _p$ and let $F/F_0$ be an unramified quadratic extension. Let $W_0$ be a split $F/F_0$-hermitian space of dimension $n+1$ and let $W_0^\flat$ be the perp-space of a vector $u_0\in W_0$ of unit length. Then the following identity holds for all *matching regular semi-simple* elements $\gamma \in \textrm{GL}_n(F)\times \textrm{GL}_{n+1}(F)$ and $g\in \textrm{U}(W_0^\flat )(F_0)\times \textrm{U}(W_0)(F_0)$,

1.0.1 $$\begin{aligned} {{\,\textrm{Orb}\,}}(g, \textbf{1}_{K^\flat \times K})=\omega (\gamma ){{\,\textrm{Orb}\,}}(\gamma ,\textbf{1}_{K'^\flat \times K'}). \end{aligned}$$

Here on the RHS, there appears the weighted orbital integral of the characteristic function of the natural hyperspecial compact subgroup $K'^\flat \times K'$ of $\textrm{GL}_n(F)\times \textrm{GL}_{n+1}(F)$; on the LHS there appears the orbital integral of the characteristic function of the natural hyperspecial compact subgroup $K^\flat \times K$ of $\textrm{U}(W_0^\flat )(F_0)\times \textrm{U}(W_0)(F_0)$. The first factor on the RHS is the natural transfer factor, cf. [31]; both sides only depend on the orbits of $\gamma$, resp. *g*, under natural group actions.

The FL conjecture was proved for *F* with large residue characteristic by Yun and Gordon [37], and is now proved completely: the proof by Beuzart-Plessis [2] is local; the proof in [42] (for $p\ge n+1$) is global.

In fact, an identity of this form is true for the whole spherical Hecke algebra, and is due to Leslie [20]. Let $\varphi '\in \mathcal {H} _{K^{\prime \flat }\times K'}$ be an arbitrary element in the spherical Hecke algebra for $\textrm{GL}_n(F)\times \textrm{GL}_{n+1}(F)$. Then the following identity holds for all matching regular semi-simple elements $\gamma \in \textrm{GL}_n(F)\times \textrm{GL}_{n+1}(F)$ and $g\in \textrm{U}(W_0^\flat )(F_0)\times \textrm{U}(W_0)(F_0)$,

1.0.2 $$\begin{aligned} {{\,\textrm{Orb}\,}}(g,\varphi )=\omega (\gamma ){{\,\textrm{Orb}\,}}(\gamma ,\varphi '). \end{aligned}$$

Here on the LHS appears the orbital integral of the image $\varphi$ of $\varphi '$ under the *base change homomorphism* from the spherical Hecke algebra of $\textrm{GL}_n(F)\times \textrm{GL}_{n+1}(F)$ to the spherical Hecke algebra of $\textrm{U}(W_0^\flat )(F_0)\times \textrm{U}(W_0)(F_0)$. The second factor on the RHS is the weighted orbital integral of $\varphi '$. The method of proof of [20] is ultimately global.

The third author proposed a relative trace formula approach to the *arithmetic* Gan–Gross–Prasad conjecture. In this context, he formulated the arithmetic fundamental lemma (AFL) conjecture [40]. The AFL relates the special value of the derivative of an orbital integral to an arithmetic intersection number on a Rapoport-Zink formal moduli space (RZ space) of *p*-divisible groups attached to a unitary group. The AFL conjecture is an identity of the following form. Let $W_1$ be a non-split $F/F_0$-hermitian space of dimension $n+1$ and let $W_1^\flat$ be the perp-space of a vector $u_1\in W_1$ of unit length. Then the following identity holds for all matching regular semi-simple elements $\gamma \in \textrm{GL}_n(F)\times \textrm{GL}_{n+1}(F)$ and $g\in \textrm{U}(W_1^\flat )(F_0)\times \textrm{U}(W_1)(F_0)$,

1.0.3 $$\begin{aligned} 2\langle g\Delta , \Delta \rangle _{\mathcal {N} _{n, n+1}}\cdot \log q=- \omega (\gamma )\partial \!\operatorname {Orb}(\gamma , {{\textbf{1}}}). \end{aligned}$$

Here the second factor on the RHS is the special value of the derivative of the weighted orbital integral of the unit element in the spherical Hecke algebra $\mathcal {H} _{K^{\prime \flat }\times K'}$. On the LHS appears the intersection number of the diagonal cycle $\Delta$ of the product RZ-space $\mathcal {N} _{n, n+1}=\mathcal {N} _n\times \mathcal {N} _{n+1}$ with its translate under the automorphism of $\mathcal {N} _{n, n+1}$ induced by *g*. Here, for any *n*, $\mathcal {N} _n$ is the moduli space of framed *basic* principally polarized *p*-divisible groups with action of $O_F$ of signature $(1, n-1)$.

The AFL conjecture is now known to hold for any odd prime *p*, cf. Zhang [42], Mihatsch–Zhang [26], Zhang [43]. These proofs are global in nature. Local proofs of the AFL are known for $n=1,2$ (Zhang [40]), and for minuscule elements (He–Li–Zhu [14]).

The aim of the present paper is to propose a variant of the AFL conjecture in the spirit of Leslie’s result on the FL, where the unit element in the spherical Hecke algebra is replaced by an arbitrary element $\varphi '\in \mathcal {H} _{K^{\prime \flat }\times K'}$. The proposed formula takes the following form,

1.0.4 $$\begin{aligned} 2\langle g\Delta , \mathbb {T} _{\varphi }(\Delta )\rangle _{\mathcal {N} _{n, n+1}}\cdot \log q=- \omega (\gamma )\partial \!\operatorname {Orb}(\gamma , \varphi '). \end{aligned}$$

The new feature compared to the AFL conjecture (1.0.3) for the unit element is the appearance of the Hecke operator $\mathbb {T} _{\varphi }$ on the LHS, and the definition of such Hecke operators is one of the main issues of the present paper, see below.

The AFL conjecture comes, as usual, in a homogeneous version (as stated above) and an inhomogeneous version. However, in contrast to the case of the unit element, these two versions are not equivalent: the homogeneous version implies the inhomogeneous version but not conversely. It is conceivable that the inhomogeneous version is easier to prove in some cases.

As evidence for this conjecture, we prove it in the case $n=1$ (in this case, the homogeneous version and the inhomogeneous version are easily seen to be equivalent).

### Theorem 1.0.1

The AFL formula (1.0.4) holds for $n=1$.

The proof is local, by explicit calculation of both sides of the formula and resembles the proof of the AFL in cases of low rank in [40]. On the geometric side we exploit the fact that, in the particular case $n=1$, the Hecke operators are induced by explicit geometric correspondences which are finite and flat. Another ingredient is the theory of quasi-canonical divisors on $\mathcal {N} _2$ in the sense of [17]. On the analytic side, we also give a purely local proof of the FL for the whole Hecke algebra (Leslie’s theorem) in this case.

Our definition of Hecke operators (in K-theory) is based on the fact that there is a presentation of the spherical Hecke algebra of the unitary group as a polynomial algebra. The basis elements of this presentation can be chosen to be decomposed into a product (i.e., convolution) of *intertwining Hecke functions*. Here an intertwining Hecke function in the Iwahori Hecke algebra for a fixed Iwahori subgroup is a function of the form $\textbf{1}_{KK'}$ for parahoric subgroups $K, K'$ stabilizing a facet in the alcove in the Bruhat–Tits building corresponding to the Iwahori subgroup^1^. For these elements it is possible to define integral models of Hecke correspondences. Indeed, we can naturally define a geometric correspondence between two RZ spaces for parahorics $K,K'$ as above, a diagram of RZ spaces at parahoric levels *K*, $K'$, $K\cap K'$,1.0.5In fact, for our purposes, it suffices to consider intertwining Hecke correspondences of the form $\textbf{1}_{K K'}$ and $\textbf{1}_{K' K}$, where $K'$ is a maximal parahoric and *K* is the hyperspecial vertex in the fixed alcove defining the spherical Hecke algebra. Hecke operators for general elements in the spherical Hecke algebra, in the sense of maps on K-groups, are then defined in three steps. The basis elements, also called *atomic elements*, can be written in the form $\phi _{K'}={{\,\textrm{vol}\,}}(K')^{-1}  \textbf{1}_{K K'}*\textbf{1}_{K' K}$, where $K'$ is a maximal parahoric subgroup corresponding to a vertex in the fixed alcove, and this defines the corresponding Hecke operator. For a monomial in the atomic elements, the corresponding Hecke operator is defined as a product of atomic Hecke operators. The general case is obtained by linear combinations. However, at this point arises a highly non-trivial problem: the definition of monomial Hecke operators presupposes that the Hecke operators corresponding to different $\phi _{K'}$ commute. We conjecture that this is indeed true but at the moment our formulation of the AFL formula (1.0.4) is contingent on the solution of this conjecture. More precisely, without this conjecture, the formulation of the AFL conjecture becomes somewhat awkward (cf. Remark 6.1.5), unless $\varphi$ is a power of an atomic element (but even this instance of the AFL formula may be interesting to prove).

The idea of defining Hecke operators as linear combinations of products of certain distinguished Hecke operators also appears in the work of Li–Mihatsch on the linear ATC [23]. In their case, the projection maps $\pi _1$ and $\pi _2$ are finite and flat, and the same is true for compositions of distinguished Hecke correspondences. This implies that their Hecke operators are induced by explicit geometric Hecke correspondences. This also allows them to pass to the generic fiber to prove the necessary commutativity statement in their context.

In our case the projection maps $\pi _1$ and $\pi _2$ are usually not flat and the composition of such correspondences, in the sense of maps on K-groups, is not induced by the composition of geometric correspondences. This discrepancy between compositions of geometric correspondences and K-group correspondences would disappear if instead of usual (*classical*) formal schemes we had used *derived* formal schemes in the definition of geometric correspondences. In this sense, our definition of Hecke operators is a “shadow” of a more sophisticated definition (which remains to be developed). By avoiding derived schemes, we forgo the possibility of defining our Hecke operators in terms of geometric Hecke correspondences.^2^ However, it is unclear to us whether such a more sophisticated definition can be helpful in resolving the commutativity conjecture mentioned above. Relatedly, it seems that the more sophisticated definition of Hecke correspondences transfers to the global context of integral models of Shimura varieties for $\textrm{GU}(1, n-1)$ but the relation to the classical Hecke correspondences in the generic fiber is unclear.

Let us compare our construction of Hecke operators with variants in the literature; indeed, the construction of integral Hecke correspondences and their induced Hecke operators on cohomology, resp. cycle groups, resp. K-groups is a well-known problem in various contexts. An example, in the context of integral models of Shimura varieties, occurs in the proof of the Eichler-Shimura congruence relation, comp. Faltings–Chai [9] and Bültel–Wedhorn [4], Koskivirta [16], Lee [19], Wedhorn [35]. Specifically, in the case of the Siegel moduli space with hyperspecial level at *p*, one considers simply the space of all isogenies of *p*-power degree and then isolates inside it a subspace that can be analyzed for the purpose at hand (note that the space of all isogenies of *p*-power degree is an unwieldy object that is hard to control). In the function field context, the problem of defining integral Hecke operators (in cohomology) is addressed by Lafforgue [18] by using his *excursion operators* (see loc. cit., Prop. 6.2); in this context, he also solves a commutativity problem (see loc. cit., Lem. 10.1, equ. (10.4)). Let us also mention the recent paper by Fakhrudin–Pilloni [8], in which they aim to define Hecke operators for automorphic vector bundles on *p*-integral models of Shimura varieties with hyperspecial level at *p*. Translated to our language of RZ spaces, they consider correspondences given by diagrams (1.0.5), where *K* and $K'$ are hyperspecial and conjugate under an auxiliary group (in their case, a group of unitary or symplectic similitudes). It should be pointed out that such diagrams exist only rarely: in the case of the symplectic group, there is precisely one such diagram (*K* is the stabilizer of a selfdual lattice and $K'$ is the stabilizer of a lattice selfdual up to a scalar), and similarly in the case of unitary groups considered here in the even rank case when *K* is the stabilizer of a selfdual lattice and $K'$ is the stabilizer of a lattice selfdual up to a scalar; in the case of the general linear group, all pairs $K, K'$ of hyperspecial subgroups corresponding to vertices in a fixed alcove give such diagrams. On the other hand, in [28, Sect. 7] Pilloni defines more general automorphic vector bundle Hecke operators for $\textrm{GSp}_4$ in a way somewhat similar to ours, via intertwining Hecke operators. It is interesting to note that in the context of [8], there is also a commutativity conjecture of Hecke operators [8, Rem. 7.6]; however, there seems to be no direct relation to our conjecture above (but maybe a solution to one of the problems can give indications for a solution to the other problem). We also note that a function field analog has been considered by Yun and the third author (cf. [38, Prop. 5.10] and [39, Prop. 3.14]), where they consider the moduli space of $\textrm{GL}_2$-shtukas (with an arbitrary number of legs) and construct Hecke correspondences for a natural basis (as a vector space) of the spherical Hecke algebra. They show a commutativity statement using crucially an equidimensionality result (cf. [38, Lem. 5.9] and [39, Lem. 3.13]), which is in turn proved via constructions closely related to the Geometric Satake isomorphism. Another attempt at defining integral Hecke correspondences occurs for RZ spaces in [32, Chap. 4]. That definition suffers from several drawbacks, the most serious being that the projection morphisms may not be proper and not surjective, cf. [32, remark after Prop. 4.44].

Why is it of interest to extend the AFL conjecture from the unit element to all elements in the spherical Hecke algebra? The reason that in the proof of the global Gan–Gross–Prasad conjecture (e.g., [3, 41]) one only considers the FL for the unit element is the density theorem of Ramakrishnan [29]. It allows one to avoid the Jacquet–Rallis fundamental lemma for the full spherical Hecke algebra at inert places. However, such a density result is not available for the orthogonal group, in which case we need necessarily to consider the full Hecke algebra. It should be pointed out, however, that at present we do not have a formulation of an FL conjecture or an AFL conjecture in the case of the orthogonal group. Another motivation comes from the consideration of the *p*-adic height pairing of arithmetic diagonal cycles, as in on-going work of Disegni and the third author [6]. Here it is necessary to consider all Hecke correspondences at *p*-adic places. This is one of the reasons, why in [6] it is assumed that all *p*-adic places are split in the quadratic extension of global fields $F/F_0$. When there are inert *p*-adic places, it will be necessary to consider Hecke correspondences at inert places and the situation of the present paper becomes relevant. One may even need to consider the more complicated case of the Iwahori level Hecke algebra. In fact, in Disegni’s work on the *p*-adic Gross–Zagier formula for Shimura curves in the inert case [5], a crucial ingredient are Hecke correspondences for arbitrarily deep level.

One spin-off of the consideration of the AFL conjecture for the spherical Hecke algebra is that it naturally leads to the following question, also partly motivated by Disegni’s work. Namely, one may ask whether a function in the spherical Hecke algebra is determined by its first derivatives of orbital integrals over regular semi-simple elements. To put this into context, it should be pointed out that a function in the spherical Hecke algebra is determined by its orbital integrals over regular semi-simple elements, cf. Proposition 8.1.1. Experimental evidence points to the fact that these two questions have quite distinct answers. Indeed, we conjecture that there is an abundance of functions with vanishing first derivatives of orbital integrals, in the following precise form.

Let $G'_{\textrm{rs}, W_1}$ denote the open subset of $\textrm{GL}_n(F)\times \textrm{GL}_{n+1}(F)$ consisting of regular semisimple elements matching with elements in the non-quasi-split unitary group $\textrm{U}(W_1^\flat )(F_0)\times \textrm{U}(W_1)(F_0)$.

### Conjecture 1.0.2

The map

$$\begin{aligned} \partial \!\operatorname {Orb}: \mathcal {H} _{K^{\prime \flat }\times K'}\rightarrow C^\infty (G'_{\textrm{rs}, W_1}) \end{aligned}$$

has a large kernel, in the sense that the kernel generates the whole ring $\mathcal {H} _{K^{\prime \flat }\times K'}$ as an ideal (note that this kernel is only a vector subspace rather than an ideal). Similarly, the map defined by the intersection numbers, ${{\,\mathrm{\textrm{Int}}\,}}: \mathcal {H} _{K^\flat \times K}\rightarrow C^\infty (G'_{\textrm{rs},W_1})$, has a large kernel.

The conjecture is somewhat speculative and we give several weaker variants of it. We confirm this conjecture in the case $n=1$.

### Theorem 1.0.3

Conjecture 1.0.2 holds when $n=1$.

However, even without spin-offs, the arithmetic fundamental lemma for the entire spherical Hecke algebra is an interesting problem of its own, which may turn out to be quite difficult. Its solution might yield additional insight into the nature of Rapoport-Zink spaces and their special cycles. It might also be a good testing ground for applying derived algebraic geometry in an unequal characteristic situation, after its success in the equal characteristic counterpart, comp. [10]. It would also be interesting to consider Hecke correspondences for other RZ spaces.

Since the arithmetic fundamental lemma for the entire spherical Hecke algebra seems so difficult, it may be instructive to prove it in special cases. We already mentioned its inhomogeneous version. Another possible simplification may occur for Hecke functions of the form $\varphi '=\textbf{1}_{K^{\prime \flat }}\otimes f'$, where $f'\in \mathcal {H} _{K'}$. Yet another simplification may occur for atomic elements. The statement for Hecke functions $\varphi '$ with base change of the form $\varphi =f\otimes \textbf{1}_{K}$, resp. $\varphi =\textbf{1}_{K^{\flat }}\otimes f'$, where $f\in \mathcal {H} _K$, resp. $f'\in \mathcal {H} _{K^\flat }$ is an atomic element, are of key importance in our paper [21].

We note that our procedure is based on integral models of Hecke correspondences for certain elements that are not contained in the spherical Hecke algebra (the function $\textbf{1}_{KK'}$ is usually not spherical). Now $\textbf{1}_{KK'}$ is contained in the Iwahori Hecke algebra (corresponding to a fixed chamber containing the facets corresponding to *K* and $K'$), and it would be interesting to see how large a subalgebra they generate, in order to define the LHS of (1.0.4) for a wider class of functions $\varphi$. On the other hand, at this moment we do not know natural candidates of smooth transfers (in the sense of Jacquet–Rallis) for functions not in the spherical Hecke algebra, so we have no immediate use for Arithmetic Transfer conjectures for all integral Hecke correspondences that we construct in this way.

In view of the global nature of the proof of Leslie’s theorem and of the various FL statements for full Hecke algebras in the Langlands program, it is natural to speculate that a proof of our AFL conjecture would necessarily require a global input. It seems that the most promising approach is to study the global *p*-adic height pairing of Nekovář, which satisfies a “modularity" condition, in the sense that the action of the Hecke algebra on this height pairing factors through an action on automorphic forms. One may leverage this modularity to deduce a version of the (global) relative trace formula identities for the *full Hecke algebra*, from the a priori weaker result for a *partial Hecke algebra* (i.e., locally the unit element at inert place and arbitrary at split places). One may then hope to deduce from such global identities the AFL identity (1.0.4). The aforementioned work of Disegni and the third author on *p*-adic height pairings [6] involves the projection to the *ordinary part*; this is an obstacle to pushing through this proof strategy.

The layout of the paper is as follows. After a notation section, we recall in Sect. 3 the set-up and review the formulation of the Jacquet–Rallis transfer and Fundamental Lemma for the full spherical Hecke algebra. In Sect. 4 we define atomic Hecke functions and exhibit the spherical Hecke algebra as the polynomial algebra of the atomic elements. In Sect. 5 we define various Rapoport–Zink spaces with certain parahoric levels, and use them to define Hecke correspondences. We then formulate the commutativity conjecture. In Sect. 6 we state the AFL conjecture for the spherical Hecke algebra, and in Sect. 7 we prove it in the case $\textrm{U} (1)\times \textrm{U} (2)$. In Sect. 8 we formulate a conjecture on the abundance of spherical Hecke functions with identically vanishing first derivative of orbital integrals. In Sect. 9 we collect a few facts on the general theory of correspondences.

We thank Tony Feng, Benjamin Howard and Andreas Mihatsch for their help. We thank SLMath for its hospitality to all three of us during the Spring 2023 semester on “Algebraic cycles, L-values, and Euler systems" when part of this work was done. We also thank the referee for his excellent work.

## Notations

Let $p>2$ be a prime. Let $F_0$ be a finite extension of $\mathbb {Q}_p$, with ring of integers $O_{F_0}$, residue field $k=\mathbb {F}_q$ of size *q*, and uniformizer $\varpi$. Let *F* be the unramified quadratic extension of $F_0$, with ring of integers $O_{F}$ and residue field $k_F$. Let $\sigma$ be the nontrivial Galois automorphism of $F/F_0$. Fix $\delta \in O_F^\times$ such that $\sigma (\delta )=-\delta$. Let ${\textrm{val}}:F\rightarrow \mathbb {Z} \cup \{\infty \}$ be the valuation on *F*. Let $|\cdot |_F:F\rightarrow \mathbb {R}_{\ge 0}$ (resp. $|\cdot |: F_0\rightarrow \mathbb {R}_{\ge 0}$) be the normalized absolute value on *F* (resp. $F_0$). Let $\eta =\eta _{F/F_0}: F_0^\times \rightarrow \{\pm 1\}$ be the quadratic character associated to $F/F_0$. We let $\widetilde{\eta }: F^\times \rightarrow \{\pm 1\}$ be the unique unramified quadratic character extending $\eta$. Let ${\breve{F}}$ be the completion of the maximal unramified extension of *F*, and ${O_{\breve{F}}}$ its ring of integers, and ${\bar{k}}$ its residue field.

A $F/F_0$-hermitian space *W* is called *split* if there exists a Witt basis; otherwise *W* is called *non-split*. Equivalently, *W* is split if $\eta _{F/F_0}((-1)^{\frac{n(n-1)}{2}}\det (W))=1$. For a vector $u\in W$ we denote by $\langle {u} \rangle$ (resp. $\langle {u} \rangle _F$) the $O_F$-submodule (resp. the *F*-submodule) generated by *u*.

For an algebraic variety *X* over $F_0$, we use the notation $C_0^\infty (X)$ for $C_0^\infty (X(F_0))$.

## FL for the full spherical Hecke algebra

In this section we review the formulation of the Jacquet–Rallis transfer and Fundamental Lemma for the full spherical Hecke algebra.

### Groups

We recall the group-theoretic setup of [30, Sect. 2] in both homogeneous and inhomogeneous settings. Let $n\ge 1$. In the homogeneous setting, set

3.1.1 $$\begin{aligned} G':={{\,\textrm{Res}\,}}_{F/F_0}(\textrm{GL}_{n}\times \textrm{GL}_{n+1}), \end{aligned}$$

a reductive algebraic group over $F_0$. Let *W* be a $F/F_0$-hermitian space of dimension $n+1$. Fix $u\in W$ a non-isotropic vector (the *special vector*), and let $W^\flat =\langle u\rangle ^\perp$. Set

3.1.2 $$\begin{aligned} G_W=\textrm{U}(W^\flat )\times \textrm{U}(W), \end{aligned}$$

a reductive algebraic group over $F_0$. We have the notion of a *regular semi-simple element*, for $\gamma \in G'(F_0)$ and for $g\in G_W(F_0)$. The notions of regular semi-simple elements are with respect to the action of the reductive algebraic group over $F_0$,

$$\begin{aligned} H'_{1, 2}=H_1'\times H_2':={{\,\textrm{Res}\,}}_{F/F_0} (\textrm{GL}_{n})\times ( \textrm{GL}_{n}\times \textrm{GL}_{n+1}) \end{aligned}$$

on $G'$, resp., of $H_{1, 2}=\textrm{U}(W^\flat )\times \textrm{U}(W^\flat )$ on $G_W$. The sets of regular semi-simple elements are denoted by $G'(F_0)_\textrm{rs}$ and $G_W(F_0)_\textrm{rs}$ respectively. We choose a basis of *W* by first choosing a basis of $W^\flat$ and then adding the special vector as the last basis vector. This then gives an identification of $G_W(F)$ with $G'(F_0)$, and defines the notion of *matching* $\gamma \leftrightarrow g$ between regular semi-simple elements of $G_W(F_0)$ and $G'(F_0)$, cf. [30, Sect. 2].

In the inhomogeneous setting, recall the symmetric space

3.1.3 $$\begin{aligned} S = S_{n+1}:= \{ g \in {{\,\textrm{Res}\,}}_{F/F_0}\textrm{GL}_{n+1} \mid g \overline{g} = 1_{n+1}\} \end{aligned}$$

and the map $r: {{\,\textrm{Res}\,}}_{F/F_0}\textrm{GL}_{n+1}\rightarrow S$ given by $g\mapsto \gamma =g\overline{g}^{-1}$, which induces an isomorphism

$$\begin{aligned} ({{\,\textrm{Res}\,}}_{F/F_0}\textrm{GL}_{n+1})/\textrm{GL}_{n+1}\simeq S. \end{aligned}$$

We have the notion of a *regular semi-simple element*, for $\gamma \in S(F_0)$ and for $g\in \textrm{U}(W)(F_0)$ and, after the choice of a basis of *W* as above, the notion of *matching* $\gamma \leftrightarrow g$. The notions of regular semi-simple elements are with respect to the conjugation actions of $H':=\textrm{GL}_{n}$ on *S*, resp., of $H:=\textrm{U}(W^\flat )$ on $\textrm{U}(W)$. The sets of regular semi-simple elements are denoted by $S(F_0)_\textrm{rs}$ and $\textrm{U}(W)(F_0)_\textrm{rs}$ respectively.

### Orbital integrals

We recall the orbital integrals in both homogeneous and inhomogeneous settings, following [30, Sect. 5]. In the homogeneous setting, for $\gamma \in G'(F_0)_\textrm{rs}$, a function $\varphi '\in C^\infty _0(G')$ and a complex parameter $s\in \mathbb {C}$, we define

3.2.1 $$\begin{aligned} {{\,\textrm{Orb}\,}}(\gamma , \varphi ', s):= \int _{H_{1,2}'(F_0)} \varphi '(h_1^{-1}\gamma h_2) |\det h_1|_F^s \eta (\det h_2)\, \textrm{d}h_1\, \textrm{d}h_2, \end{aligned}$$

where we use the normalized Haar measures on $H_1'(F_0)$ and $H_2'(F_0)$ (such that the obvious hyperspecial maximal compact open groups have measure one) and the product Haar measure on $H_{1,2}'(F_0) = H_1'(F_0) \times H_2'(F_0)$. We further define the value and derivative at $s=0$,

3.2.2 $$\begin{aligned} {{\,\textrm{Orb}\,}}(\gamma , \varphi '):= {{\,\textrm{Orb}\,}}(\gamma , \varphi ', 0) \quad \text {and}\quad \partial \!\operatorname {Orb}(\gamma , \varphi '):= \frac{\textrm{d}}{\textrm{d}s} \Big |_{s=0} {{\,\textrm{Orb}\,}}({\gamma }, \varphi ',s).\nonumber \\ \end{aligned}$$

The integral defining ${{\,\textrm{Orb}\,}}(\gamma ,\varphi ',s)$ is absolutely convergent, and (upon introducing a suitable transfer factor) ${{\,\textrm{Orb}\,}}(\gamma , \varphi ')$ and $\partial \!\operatorname {Orb}(\gamma , \varphi ')$ depend only on the orbit of $\gamma$.

Now we turn to the inhomogeneous setting. For $\gamma \in S(F_0)_\textrm{rs}$, a function $\phi \in C_c^\infty (S)$, and a complex parameter $s\in \mathbb {C}$, we introduce the *weighted orbital integral*

3.2.3 $$\begin{aligned} {{\,\textrm{Orb}\,}}(\gamma ,\phi ', s) := \int _{H'(F_0)}\phi '(h^{-1}\gamma h)|\det h |^s \eta (\det h) \, \textrm{d}h , \end{aligned}$$

as well as the value and derivative at $s=0$,

$$\begin{aligned} {{\,\textrm{Orb}\,}}(\gamma ,\phi '):= {{\,\textrm{Orb}\,}}(\gamma ,\phi ', 0) \quad \text {and}\quad \partial \!\operatorname {Orb}(\gamma ,\phi '): = \frac{\textrm{d}}{\textrm{d}s} \Big |_{s=0} {{\,\textrm{Orb}\,}}(\gamma , \phi ',s). \end{aligned}$$

As in the homogeneous setting, the integral defining ${{\,\textrm{Orb}\,}}(\gamma ,\phi ', s)$ is absolutely convergent, and (upon introducing a suitable transfer factor) ${{\,\textrm{Orb}\,}}(\gamma , \phi ')$ and $\partial \!\operatorname {Orb}(\gamma , \phi ')$ depend only on the orbit of $\gamma$.

There are also corresponding orbital integrals on the unitary side. In the homogeneous setting, for $g\in G_{W}(F_0)_\textrm{rs}$ and a function $\varphi \in C^\infty _0(G_{W})$, we define

3.2.4 $$\begin{aligned} {{\,\textrm{Orb}\,}}(g, \varphi ):= \int _{H_{1,2}(F_0)} \varphi (h_1^{-1}\gamma h_2) \, \textrm{d}h_1\, \textrm{d}h_2. \end{aligned}$$

When $W^\flat$ is split, we normalized the Haar measure on $\textrm{U}(W^\flat )$ such that the hyperspecial maximal compact subgroups get measure one, and take the product Haar measure on $H_{1,2}(F_0)$. In the inhomogeneous setting, we set for $g\in \textrm{U}(F_0)_\textrm{rs}$ and a function $\phi \in C_c^\infty (U(W))$,

3.2.5 $$\begin{aligned} {{\,\textrm{Orb}\,}}(g, \phi ):= \int _{\textrm{U}(F_0)} \phi (h^{-1} g h) \, \textrm{d}h. \end{aligned}$$

### Matching and transfer

Let $W_0$, $W_1$ be representatives of the two isomorphism classes of $F/F_0$-hermitian spaces of dimension $n+1$. We assume $W_0$ to be split. Take the special vectors $u_0\in W_0$ and $u_1\in W_1$ to have the same norm (not necessarily a unit). We also choose bases of $W_0$ and $W_1$ as above. Then matching defines bijections of regular semisimple orbits3.3.1in the homogeneous setting, and3.3.2in the inhomogeneous setting, cf. [30, Sects. 2.1, 2.2].

Then associated to a transfer factor $\omega _{G'}: G'(F_0)_\textrm{rs} \rightarrow \mathbb {C}^\times$ we have the notion of *transfer* between functions $\varphi '\in C_c^\infty (G')$ and pairs of functions $(f_0,f_1)\in C_c^\infty (G_{W_0})\times C_c^\infty (G_{W_1})$ ([31, Definition 2.2]). We will always use the transfer factor given by [31, (5.2)] (extrapolated in the obvious way from odd *n* to even *n*). Similarly, for a transfer factor $\omega _S: S(F_0)_\textrm{rs} \rightarrow \mathbb {C}^\times$ we have the notion of *transfer* between functions $\phi '\in C_c^\infty (S)$ and pairs of functions $(f_0,f_1)\in C_c^\infty (\textrm{U}(W_0))\times C_c^\infty (\textrm{U}(W_1))$ ( [31, Definition 2.4]) We will always use the transfer factor given by [31, (5.5)] (again extrapolated to all *n*).

### Satake isomorphism and base change

Let ${K'}=K'_n=\textrm{GL}_n(O_{F})$. We denote by $\mathcal {H}_{K'}=\mathbb {Q} [K'_n\backslash \textrm{GL}_n(F)/K'_n]$ the Hecke algebra with coefficients in $\mathbb {Q}$ of $\textrm{GL}_n$. Haar measures are chosen such that maximal compact subgroups have measure one.

We first recall the Satake isomorphism for $\textrm{GL}_n$ over *F*. Let $T\subseteq \textrm{GL}_n$ be the diagonal torus. The cocharacter group $X_*(T)$ is a free abelian group generated by $\{\mu _1,\ldots , \mu _n\}$, where $\mu _i$ is the injection in the *i*-th factor. For $\mu \in X_*(T)$, denote by $[\mu ]$ the corresponding element in the group algebra $\mathbb {C}[X_*(T)]$. For $1\le i\le n$, define

3.4.1 $$\begin{aligned} x_i:=[\mu _i]\in \mathbb {C}[X_*(T)]. \end{aligned}$$

We identify $\mathbb {C}[X_*(T)]$ with $\mathbb {C}[T(F)/T(O_F)]$ by sending $\lambda \in X_*(T)$ to the monomial diagonal matrix $\varpi ^\lambda$. Let $\sigma _i$ be the degree *i* elementary symmetric polynomial in $\{x_1,\ldots ,x_n\}$, which corresponds to the sum of the elements in the $S_n$-orbit of $\varpi ^{(1^i, 0^{n-i})}T(O_F)$. Then the Satake transform gives an isomorphism of algebras3.4.2It sends the minuscule function $\textbf{1}_{{K'}\varpi ^{(1^i, 0^{n-i})}{K'}}$ to $q_F^{i(n-i)/2}\sigma _i=q^{i(n-i)}\sigma _i$. Note that since the modulus function $\delta _B^{\frac{1}{2}}$ takes values in $\mathbb {Q}$, the homomorphism (3.4.2) is defined over $\mathbb {Q}$.

Next we recall the Satake isomorphism for the unramified unitary group. Let now $W_0$ be a split $F/F_0$-hermitian space of dimension *n*. Let $m=\lfloor n/2\rfloor$. We choose a basis of $W_0$ such that the hermitian form is given by the antidiagonal unit matrix. Let $\Xi \subset W_0$ be the standard lattice, which is self-dual, and let $K\subset \textrm{U}(W_0)(F_0)$ be its stabilizer. Let $\mathcal {H}_K=\mathbb {Q} [K\backslash \textrm{U}(W_0)(F_0)/K]$ be the Hecke algebra with coefficients in $\mathbb {Q}$ of $\textrm{U}(W_0)$. We recall the Satake isomorphism for $\textrm{U}(W_0)$. Let *A* be the maximal split diagonal torus in $\textrm{U}(W_0)$. With the chosen basis, we can identify *A* with the torus consisting of diagonal elements of the form ${{\,\textrm{diag}\,}}(x_1,\ldots ,x_m,1_{n-2m}, x_{m}^{-1},\ldots , x_1^{-1})$ in $\textrm{U}(W_0)$. For $1\le s\le m$, let $\nu _s=\mu _s-\mu _{n+1-s}\in X_*(A)$ be the cocharacter of *A* sending *x* to ${{\,\textrm{diag}\,}}(1,\ldots ,x_s=x,\ldots ,1,1_{n-2\,m}, 1, \ldots , x_{n+1-s}=x^{-1},\ldots , 1)$. For $1\le s\le m$ we define

$$\begin{aligned} y_s:=[\nu _{s}]+[-\nu _{s}]\in \mathbb {C}[X_*(A)]. \end{aligned}$$

Let $\mathfrak {s}_s$ be the degree *s* elementary symmetric polynomial in $\{y_1,\ldots ,y_m\}$. Then the Satake transform gives an isomorphism of algebras3.4.3where $W_n\simeq (\mathbb {Z}/2 \mathbb {Z})^m\rtimes S_m$ is the Weyl group of $A(F_0)$ in $\textrm{U}(W_0)(F_0)$. In particular, $\mathcal {H}_K$ is a polynomial algebra. Analogous to the case of $\textrm{GL}_n$ (over *F*), the modulus function $\delta _B^{\frac{1}{2}}$ takes values in $\mathbb {Q}$, hence the homomorphism (3.4.3) is defined over $\mathbb {Q}$.

We have an algebra homomorphism, called the base change homomorphism,

3.4.4 $$\begin{aligned} \textrm{BC}:\mathcal {H}_{K'}\rightarrow \mathcal {H}_K,\quad \varphi '\mapsto \textrm{BC}(\varphi '). \end{aligned}$$

The homomorphism $\textrm{BC}$ is characterized by the identity

$$\begin{aligned} \textrm{trace}\, \Pi (\varphi ')= \textrm{trace}\, \pi ( \textrm{BC}(\varphi ')), \end{aligned}$$

where $\Pi$ denotes the base change of $\pi$, for all unramified representations $\pi$ of $\textrm{U}(W_0)$.

An alternative description is in terms of a morphism of the Langlands dual tori $\widehat{A}\rightarrow \widehat{T}$. More precisely, we may identify $\widehat{T}$ with $(\mathbb {C} ^\times )^n$, sending the character $\mu _i\in X^*(\widehat{T})=X_{*}(T)$ of $\widehat{T}$ to the *i*-th coordinate $\alpha _i$ on $(\mathbb {C} ^\times )^n$. We may identify $\widehat{A}$ as the subtorus $(\mathbb {C} ^n)^{\textrm{unit}}$ of $\widehat{T}= (\mathbb {C} ^\times )^n$, where $(\mathbb {C} ^n)^{\textrm{unit}}$ denotes the space of unitary parameters in $\mathbb {C} ^n$,

3.4.5 $$\begin{aligned} \begin{aligned} (\mathbb {C} ^n)^{\textrm{unit}}=\{ \alpha =(\alpha _1,\ldots ,\alpha _n)\in \mathbb {C}^n\mid \alpha _i\alpha _{n+1-i}=1\,\, \forall i,\\ \text {and } \alpha _{m+1}=1 \text { if } n=2m+1\}, \end{aligned} \end{aligned}$$

such that the character $\nu _i\in X^*(\widehat{A})=X_{*}(A)$ of $\widehat{A}$ corresponds to the *i*-th coordinate $\alpha _i$. In particular, the element $y_s=[\nu _{s}]+[-\nu _{s}]\in \mathbb {C}[X_*(A)]=\mathbb {C}[X^*(\widehat{A})]$ corresponds to $\alpha _s+\alpha _{s}^{-1}$ as regular functions on $\widehat{A}$. The morphism $\widehat{A}\rightarrow \widehat{T}$ is then the natural inclusion

$$\begin{aligned} \textrm{bc}:(\mathbb {C} ^n)^{\textrm{unit}}\hookrightarrow (\mathbb {C} ^\times )^n, \end{aligned}$$

and the base change homomorphism is characterized by

$$\begin{aligned} \textrm{Sat}(\varphi ')(\textrm{bc}(\alpha ))=\textrm{Sat}(\textrm{BC}(\varphi '))(\alpha ). \end{aligned}$$

We denote by the same symbol the induced homomorphism,

$$\begin{aligned} \textrm{BC}:\mathbb {Q} [\sigma _1,\sigma _2,\ldots ,\sigma _{n-1}, \sigma _n^{\pm }]\rightarrow \mathbb {Q} [\mathfrak {s}_1,\ldots , \mathfrak {s}_m]. \end{aligned}$$

We obtain a commutative diagram3.4.6For example, we have

$$\begin{aligned} \textrm{BC}(\sigma _1)=\left\{ \begin{array}{ll} \mathfrak {s}_1 & \text {if } n \text { is even} \\ \mathfrak {s}_1+1& \text {if } n \text { is odd.}\end{array}\right. \end{aligned}$$

### Some explicit examples

In this subsection we make a digression to give some examples of Satake transforms and the base change homomorphism. Most of it will not be used later. We use for $W_0$ the Hermitian form on the standard vector space given by an anti-diagonal matrix.

For even *t* with $0\le t\le n$, we introduce the functions

3.5.1 $$\begin{aligned} f^{[t]}:=\textbf{1}_{K\varpi ^{(1^{{t}/{2}}, 0^{n-t}, (-1)^{t/2})}K}\in \mathcal {H}_K. \end{aligned}$$

Note that these functions form a polynomial basis of $\mathcal {H} _K$. We wish to determine their Satake transforms.

We recall some results from [24]. The Langlands dual group of $\textrm{U}(W_0)$ is the semi-direct product $\textrm{GL}_n(\mathbb {C})\rtimes {{\,\textrm{Gal}\,}}(F/F_0)$. Let $A'$ be the maximal torus containing *A*. Let ${\hat{A}}'\subset \textrm{GL}_n(\mathbb {C})$ be the diagonal torus in the dual group of $\textrm{GL}_n$. Let $\chi (\rho _{n,s})$ be the restriction of the character of $\wedge ^s Std\otimes \wedge ^s Std^\vee$ to ${\hat{A}}'\times \{\sigma \}\subset \textrm{GL}_n(\mathbb {C})\rtimes {{\,\textrm{Gal}\,}}(F/F_0)$, viewed as an element in $\mathbb {Z} [X^*({\hat{A}}')]=\mathbb {Z} [X_*(A')]$. Then $\chi (\rho _{n,s})\in \mathbb {C} [X_*(A')]^{S_n, \sigma }= \mathbb {C} [X_*(A)]^{W_n}=\mathbb {C} [\mathfrak {s}_1,\ldots ,\mathfrak {s}_{m}]$. Here we refer to loc. cit. for the definition of the semi-direct product (defining the L-group of $\textrm{U}(W_0)$) and of the action of $\sigma$ on the representation space. Then [24, Lem. B.2] (the latter is also [36, Lem. 9.2.4])

$$\begin{aligned} \chi (\rho _{n,s})=\left\{ \begin{array}{ll} \sum _{j=0}^{[s/2]} \left( \begin{matrix}m-(s-2j)\\ j \end{matrix}\right) \mathfrak {s}_{s-2j}, &  n \text { even}\\ \sum _{i=0}^s \left( \begin{matrix}m-(s-i)\\ [i/2] \end{matrix}\right) \mathfrak {s}_{s-i},&n \text { odd.} \end{array}\right. \end{aligned}$$

Set

$$\begin{aligned} [n]_q=\frac{q^n-1}{q-1}, \quad [n]_q!= [n]_q [n-1]_q\cdots [1]_q, \quad \left[ \begin{matrix}n\\ m \end{matrix}\right] _q=\frac{[n]_{q}!}{[m]_{q}![n-m]_{q}!} \end{aligned}$$

The Satake transforms of $f^{[2s]}$, $1\le s\le m$, are determined by the following identity in $\mathbb {C} [\mathfrak {s}_1,\ldots , \mathfrak {s}_m]$, cf. [24, Lem. 2.6],

$$\begin{aligned} q^{s(n-s)} \chi (\rho _{n,s})=\sum _{i=0}^s \left[ \begin{matrix}n-2i\\ s -i\end{matrix}\right] _{-q} \textrm{Sat}(f^{[2i]}),\quad 1\le s\le m. \end{aligned}$$

For completeness we also recall [24, Lem. B.1.3, B.1.4]:

$$\begin{aligned} \prod _{t=1}^m(\lambda +\lambda ^{-1}+y_t)=\left\{ \begin{array}{ll} \chi (\rho _{n,m})+\sum _{i=1}^m \chi (\rho _{n,m-i})( \lambda ^i+\lambda ^{-i}), &  n \text { even}\\ \sum _{i=0}^m \chi (\rho _{n,m-i})\frac{( \lambda ^{i+1}+\lambda ^{-i})}{\lambda +1}, &  n \text { odd,} \end{array}\right. \end{aligned}$$

as an identity of finite Laurent series in $\lambda$.

#### Example 3.5.1

Taking $s=1$ we obtain

$$\begin{aligned} \left[ \begin{matrix}n\\ 1\end{matrix}\right] _{-q}\textrm{Sat}(f^{[0]})+\left[ \begin{matrix}n-2\\ 0\end{matrix}\right] _{-q}\textrm{Sat}(f^{[2]})=q^{n-1} \left\{ \begin{array}{ll} \mathfrak {s}_1, &  n\text { even},\\ \mathfrak {s}_1+1, &  n\text { odd}. \end{array}\right. \end{aligned}$$

Therefore

$$\begin{aligned} \textrm{Sat}(f^{[2]})=-[n]_{-q}+q^{n-1} \left\{ \begin{array}{ll} \mathfrak {s}_1, &  n\text { even},\\ \mathfrak {s}_1+1, &  n\text { odd}. \end{array}\right. \end{aligned}$$

Taking $s=2$ we obtain

$$\begin{aligned}  &   \left[ \begin{matrix}n\\ 2\end{matrix}\right] _{-q}\textrm{Sat}(f^{[0]})+\left[ \begin{matrix}n-2\\ 1\end{matrix}\right] _{-q}\textrm{Sat}(f^{[2]})+\left[ \begin{matrix}n-4\\ 0\end{matrix}\right] _{-q}\textrm{Sat}(f^{[4]})\\  &   \quad =q^{2(n-2)} \left\{ \begin{array}{ll} \mathfrak {s}_2+m, &  n\text { even},\\ \mathfrak {s}_2+\mathfrak {s}_1+m, &  n\text { odd}. \end{array}\right. \end{aligned}$$

We also describe some explicit functions $\varphi ^{\prime [t] }$ such that $\textrm{BC}(\varphi ^{\prime [t]})=f^{[t]}$ for even *t*. For $\varphi '\in \mathcal {H}_{K'}$, we view $\varphi '(\alpha _1,\ldots ,\alpha _n)$, where $(\alpha _1,\ldots ,\alpha _n)\in (\mathbb {C} ^n)^{\textrm{unit}}$, as a symmetric polynomial in $\alpha _i+\alpha _i^{-1}$ for $1\le i\le m$, then the resulting polynomial is nothing but $\textrm{BC}(\varphi ')$. For example,

$$\begin{aligned} \begin{aligned} \sigma _2(\alpha _1,\ldots ,\alpha _n)=\sum _{1\le i<j\le n}\alpha _i\alpha _j=&\sum _{1\le i<j\le m}(\alpha _i+\alpha _i^{-1})(\alpha _j+\alpha _j^{-1})\\&+\sum _{1\le i\le m}\alpha _i\alpha _i^{-1} (+\sum _{1\le i\le m}(\alpha _i+\alpha _i^{-1}) \text { if } n \text { is odd}). \end{aligned} \end{aligned}$$

Thus

$$\begin{aligned} \textrm{BC}(\sigma _2)= \left\{ \begin{array}{ll} \mathfrak {s}_2+m, &  n\text { even},\\ \mathfrak {s}_2+\mathfrak {s}_1+m, &  n\text { odd}. \end{array}\right. \end{aligned}$$

In general, for $1\le s\le m$, we have

$$\begin{aligned} \textrm{BC}(\sigma _s)=\chi (\rho _{n,s}). \end{aligned}$$

Thus $\{\textrm{Sat}(f^{[2s]})\}$ can be written as linear combination of $\{\textrm{BC}(\sigma _s)\}$ given by

$$\begin{aligned} \begin{pmatrix} \textrm{Sat}(f^{[0]})\\ \textrm{Sat}(f^{[2]})\\ \vdots \\ \textrm{Sat}(f^{[2m]}) \end{pmatrix}= \begin{pmatrix} \left[ {\begin{smallmatrix}n\\ 0\end{smallmatrix}}\right] _{-q} &  0 & \cdots &  0\\ \left[ {\begin{smallmatrix}n\\ 1\end{smallmatrix}}\right] _{-q} &  \left[ {\begin{smallmatrix}n-2\\ 0\end{smallmatrix}}\right] _{-q} & \cdots &  0\\ \vdots &  \vdots & \ddots & \vdots \\ \left[ {\begin{smallmatrix}n\\ m\end{smallmatrix}}\right] _{-q} & \left[ {\begin{smallmatrix}n-2\\ m-1\end{smallmatrix}}\right] _{-q} &  \cdots &  \left[ {\begin{smallmatrix}n-2m\\ 0\end{smallmatrix}}\right] _{-q} \end{pmatrix}^{-1}\cdot \begin{pmatrix} 1\\ q^{n-1}\cdot \textrm{BC}(\sigma _1)\\ \vdots \\ q^{m(n-m)}\cdot \textrm{BC}(\sigma _m) \end{pmatrix} \end{aligned}$$

### The fundamental lemma, homogeneous version

We apply the preceding considerations to a split space $W_0$ of dimension $n+1$ and to $W_0^\flat =\langle u_0\rangle ^\perp$, where the special vector $u_0\in \Xi$ has unit length. We denote by $K^\flat$ the stabilizer of the selfdual lattice $\Xi ^\flat :=W_0^\flat \cap \Xi$. The Haar measures are chosen so that hyperspecial maximal open subgroups have measure one. The Hecke algebra $\mathcal {H} _{K^\flat \times K}$ for $\textrm{U}(W_0^\flat )\times \textrm{U}(W_0)$ can be identified with the tensor product of algebras $\mathcal {H}_{K^\flat }\otimes _\mathbb {Q} \mathcal {H}_K$. The analogous facts hold for the triple $(\textrm{GL}_n, \textrm{GL}_{n+1}, \textrm{GL}_n\times \textrm{GL}_{n+1})$ and the standard open compact subgroups $({K'}^\flat , {K'}, {K'}^\flat \times {K'})$. We use the same symbol $\textrm{BC}$ for the analogous algebra homomorphisms. More precisely, we have

$$\begin{aligned}  &   \textrm{BC}_{n}: \mathcal {H} _{K^{\prime \flat }}\rightarrow \mathcal {H} _{K^{ \flat }}, \quad \textrm{BC}_{n+1}: \mathcal {H} _{K'}\rightarrow \mathcal {H}_K,\\  &   \textrm{BC}=\textrm{BC}_{n}\otimes \textrm{BC}_{n+1}:\mathcal {H}_{K^{\prime \flat }}\otimes _{\mathbb {Q}} \mathcal {H}_{K'}\rightarrow \mathcal {H}_{K^\flat }\otimes _{\mathbb {Q}}\mathcal {H}_K. \end{aligned}$$

We have the following fundamental lemma for the spherical Hecke algebra in the Jacquet-Rallis case. In it, we let $W_1$ be the non-split space of dimension $n+1$ and $W_1^\flat =\langle u_1\rangle ^\perp$, where the special vector $u_1$ has unit length. Also, we have chosen bases of $W_0$ and $W_1$ as usual.

#### Theorem 3.6.1

(FL for the spherical Hecke algebra (homogeneous version) (Leslie [20])) Let $\varphi '\in \mathcal {H}_{K^{\prime \flat }}\otimes \mathcal {H}_{K'}$. Then

$$\begin{aligned} (\textrm{BC}(\varphi '),0)\in C_c^\infty (G_{W_0})\times C_c^\infty (G_{W_1}) \end{aligned}$$

is a transfer of $\varphi '$. $\square$

### The fundamental lemma, inhomogeneous version

Recall the action of $\textrm{GL}_n$ on $S=S_n$, cf. (3.1.3) (except that here *n* replaces $n+1$). Let

$$\begin{aligned} \mathcal {H} _{K'_{S_n}}=\mathcal {C} ^\infty _c(S_n(F_0))^{K_n'}. \end{aligned}$$

Note that this is a module over the Hecke algebra $\mathcal {H} _{K'_n}=\mathcal {H} _{K'_n}(\textrm{GL}_n)$. We obtain a map

3.7.1 $$\begin{aligned} \textrm{act}: \mathcal {H} _{K'_n}\rightarrow \mathcal {H} _{K'_{S_n}}, \quad f'\mapsto f'*\textbf{1}_{K'_{S_n}}. \end{aligned}$$

Here $\textbf{1}_{K'_{S_n}}$ denotes the characteristic function of $K'_{S_n}=K'_n\cdot 1$.

There is an alternative description. Let $r: G'\rightarrow S_n$ be the map $g\mapsto g \overline{g}^{-1}$. We also have the map induced by integration on the fibers

3.7.2 $$\begin{aligned} r_*:\mathcal {H} _{K'_n}\rightarrow \mathcal {H} _{K'_{S_n}}, \end{aligned}$$

which sends $f'$ to $f^{' \natural }$ defined by

$$\begin{aligned} f^{' \natural }(g \overline{g}^{-1})=\int _{\textrm{GL}_n(F_0)} f'(gh)\, dh. \end{aligned}$$

Here we choose the Haar measure on $\textrm{GL}_n(F_0)$ such that ${{\,\textrm{vol}\,}}(\textrm{GL}_n(O_{F_0}))=1$. Then it is easy to check that the two maps (3.7.1) and (3.7.2) coincide, cf. [20, Lem. 3.3].

Using a theorem of Offen [27], Leslie shows that both maps factor through the base change homomorphism $\textrm{BC}=\textrm{BC}_{n}: \mathcal {H} _{K'_n}(\textrm{GL}_n)\rightarrow \mathcal {H} _{K}$ and induce an isomorphism with the Hecke algebra for the quasi-split unitary group,3.7.3cf. [20, Cor. 3.5]. We thus have the following commutative diagram,3.7.4For the inhomogeneous FL we need a twist by the algebra automorphism $\eta _\mathcal {H}: \mathcal {H} _{K'_n}\rightarrow \mathcal {H} _{K'_n}$ defined by $f\mapsto f\widetilde{\eta }$, where $\widetilde{\eta }(g)=(-1)^{v(\det (g))}$ is our fixed extension of the character $\eta$. In terms of the Satake isomorphism (3.4.2), this automorphism translates to the map $x_i\mapsto -x_i$. In particular, this shows that the map $\eta _\mathcal {H}$ is an algebra homomorphism. Via the Satake isomorphism we see that the involution $\eta _\mathcal {H}$ descends to $\mathcal {H} _K$, which we also denote by $\eta _\mathcal {H}$. We have a commutative diagramLet $i\ge 0$. We introduce the $\eta ^i$-twisted version of (3.7.3) by3.7.5where $\eta _\mathcal {H} ^i$ is the *i*-fold iterate of the automorphism $\eta _\mathcal {H}$. To have a diagram similar to (3.7.4), we introduce the $\eta ^i$-twist of $r_*$:

3.7.6 $$\begin{aligned} r^{\eta ^i}_*=r_*\circ \eta _\mathcal {H} ^i. \end{aligned}$$

Explicitly, we have

3.7.7 $$\begin{aligned} r^{\eta ^i}_*(f')(g \overline{g}^{-1})=\int _{\textrm{GL}_n(F_0)} f'(gh)\widetilde{\eta }^i(gh)\, dh. \end{aligned}$$

Then we have a commutative diagram3.7.8There is then the following inhomogeneous version of the fundamental lemma for the spherical Hecke algebra in the Jacquet-Rallis case. Here we recall that $W_0$ denotes the split hermitian space of dimension $n+1$ and $W_1$ the non-split space (therefore there is a shift of dimensions compared to above).

#### Theorem 3.7.1

(FL for the spherical Hecke algebra (inhomogeneous version) (Leslie [20])) Let $\varphi '\in \mathcal {H} _{K'_{S_{n+1}}}$. Then^3^

$$\begin{aligned} (\textrm{BC}^{\eta ^{n}}_{S_{n+1}}(\varphi '),0)\in C_c^\infty (\textrm{U}({W_0}))\times C_c^\infty (\textrm{U}({W_1})) \end{aligned}$$

is a transfer of $\varphi '$. $\square$

The inhomogeneous version is equivalent to the special case of the homogeneous version when the factor on $\mathcal {H} _{K_{n}}$ is the identity $\textbf{1}_{K'_{n}}$. To see this, we compare orbital integrals: the homogeneous version defined by (3.2.1) and the inhomogeneous one defined by (3.2.3). The following easy lemma is a combination of [30, Lem. 5.7] and [31, Lem. 14.7 (iii)].

#### Lemma 3.7.2

Let $\varphi '\in \mathcal {H}_{K^{\prime \flat }}\otimes \mathcal {H}_{K'}$ be of the form $\textbf{1}_{K'^\flat }\otimes f'$ with $f'\in \mathcal {H}_{K'}$. Then we have, for $\gamma \in S_{n+1}(F_0)_{\textrm{rs}}$ with $\gamma =r(g)=g\overline{g}^{-1}$ with $g\in \textrm{GL}_{n+1}(F)$,

$$\begin{aligned} {{\,\textrm{Orb}\,}}\bigl ((1,g),\textbf{1}_{K'^\flat }\otimes f', s\bigr )=\widetilde{\eta }^{-n}(g) {{\,\textrm{Orb}\,}}(\gamma ,r_{*}^{\eta ^n}(f'), 2s). \end{aligned}$$

Moreover, for $g=(g_1,g_2)\in G'(F_0)_\textrm{rs}$ and $\gamma =r(g_1^{-1}g_2)\in S(F_0)_\textrm{rs}$, we have

$$\begin{aligned} \omega _{G'}(g) {{\,\textrm{Orb}\,}}\bigl (g,\textbf{1}_{K'^\flat }\otimes f'\bigr )=\omega _{S}(\gamma ) {{\,\textrm{Orb}\,}}(\gamma ,r_{*}^{\eta ^n}(f')), \end{aligned}$$

and when ${{\,\textrm{Orb}\,}}\bigl (g,\textbf{1}_{K'^\flat }\otimes f'\bigr )=0$, we have

$$\begin{aligned} \omega _{G'}(g) \partial \!\operatorname {Orb}\bigl (g,\textbf{1}_{K'^\flat }\otimes f'\bigr )=2\omega _{S}(\gamma ) \partial \!\operatorname {Orb}(\gamma ,r_{*}^{\eta ^n}(f')). \end{aligned}$$

#### Proof

By definition of the orbital integral (3.2.1), we have

$$\begin{aligned} {{\,\textrm{Orb}\,}}\bigl ((1,g),\varphi ', s\bigr ) = \int _{H_{1, 2}'(F_0)}\varphi '(h_1^{-1}h_2',h_1^{-1} g h_2'') |\det h_1|_F^s \eta (h_2) \,dh_1\,dh_2'\,dh_2'', \end{aligned}$$

where $h_1\in H_1'(F_0) = \textrm{GL}_{n-1}(F)$ and $h_2=(h_2',h_2'')\in H_2'(F_0)=\textrm{GL}_{n}(F_0)\times \textrm{GL}_{n+1}(F_0)$. Here $|\cdot |_F$ is the normalized absolute value on *F*. Also $\eta (h_2)=\eta ^{n-1}(\det h_2')\eta ^{n}(\det h_2'')$. Replacing $h_1$ by $h_2'h_1$, we have

$$\begin{aligned} {{\,\textrm{Orb}\,}}\bigl ((1,g),\varphi ', s\bigr ) = \int _{H_{1, 2}'(F_0)} \varphi '\bigl (h_1^{-1},h_1^{-1}(h_2')^{-1}g h_2''\bigr ) |\det (h_2'h_1)|_F^s \eta (h_2)\,dh_1 \,dh_2'\, dh_2''. \end{aligned}$$

Now we specialize to $\varphi '=\textbf{1}_{K'^\flat }\otimes f'$. Then the above equation simplifies to

$$\begin{aligned} {{\,\textrm{Orb}\,}}\bigl ((1,g),\varphi ', s\bigr ) = \int _{H_{2}'(F_0)} f'\bigl ((h_2')^{-1}g h_2''\bigr ) |\det (h_2')|_{F_0}^{2s} \eta (h_2) \,dh_2'\, dh_2''. \end{aligned}$$

Here we have used $|a|_F=|a|_{F_0}^2$ for $a\in F_0^\times$ and this results in the extra factor 2 in the exponent. To apply the definition of $r_{*}^{\eta ^n}$, we rewrite it as

$$\begin{aligned}  &   {{\,\textrm{Orb}\,}}\bigl ((1,g),\varphi ', s\bigr ) \\  &   \quad =\widetilde{\eta }^{-n}(g) \int _{H_{2}'(F_0)} f'\bigl ((h_2')^{-1}g h_2''\bigr ) |\det (h_2')|_{F_0}^{2s} \eta (h_2')\widetilde{\eta }^n((h_2')^{-1}g h_2'') \,dh_2'\, dh_2''. \end{aligned}$$

We integrate over $h_2''\in \textrm{GL}_{n+1}(F_0)$ to obtain

$$\begin{aligned} {{\,\textrm{Orb}\,}}\bigl ((1,g),\varphi ', s\bigr ) = \widetilde{\eta }^{-n}(g)\int _{H_{2}'(F_0)} r_{*}^{\eta ^n}(f')\bigl ((h_2')^{-1}(g\overline{g}^{-}) h_2'\bigr ) |\det (h_2')|_{F_0}^{2s} \eta (h_2') \,dh_2'. \end{aligned}$$

A direct comparison with (3.2.3) completes the proof of the first identity.

For the other identities, we compare the transfer factors on $G'$ and *S*. In fact, by their definitions in [31, Sect. 2.4] and noting that $\widetilde{\eta }$ is of order two by our choice, we see that

$$\begin{aligned} \omega _{G'}(g) = \widetilde{\eta }(g_1^{-1}g_2)^n \omega _S\bigl (r(g_1^{-1}g_2)\bigr ). \end{aligned}$$

Note that our $n+1$ corresponds to *n* in loc. cit. The desired identities follow immediately. $\square$

## An alternative basis of $\mathcal {H}_K$

### An alternative basis of $\mathcal {H}_K$

We again denote by $W_0$ a split Hermitian space of dimension *n*. Let $m=[n/2]$. Fix $\Xi _0$ a self-dual lattice in $W_0$ and let $K=K_0$ be the stabilizer of $\Xi _0$. Recall that a vertex lattice in $W_0$ is a lattice $\Lambda$ such that $\Lambda \subset \Lambda ^\vee \subset \varpi ^{-1}\Lambda$. Here $\Lambda ^\vee$ is the dual lattice of $\Lambda$. The dimension $t=\Lambda ^\vee /\Lambda$ is called the type of $\Lambda$. It is an even integer with $0\le t\le n$. We fix a maximal chain of vertex lattices

4.1.1 $$\begin{aligned} \Xi _0\supset \Xi _2\supset \cdots \supset \Xi _{2m}, \end{aligned}$$

where $\Xi _t$ is a type *t* vertex lattice, for every even integer $0\le t\le n$. Let $K_t\subset \textrm{U} (W_0)$ be the stabilizer of $\Xi _t$, a special maximal parahoric subgroup. Set

4.1.2 $$\begin{aligned} \varphi _{[t,t']}=\textbf{1}_{K_t K_{t'}}\in \mathcal {H} (K_t\backslash \textrm{U} (W_0)/ K_{t'}), \end{aligned}$$

which will be called the *intertwining function* for the level $K_t,K_{t'}$. (See [24, Definition B.2.3] for the special case $t=t'=2m$.) Note that $\textbf{1}_{K_t K_{t'}}$ is the characteristic function of a subset of $\textrm{U} (W_0)$ that is not a subgroup, unless $K_t =K_{t'}$.

Note that a function $\varphi \in C_c^{\infty }(K\backslash G/K')$ induces a natural map $C_c^\infty (G/ K)\rightarrow C_c^\infty (G/ K')$ which determines the function uniquely. Explicitly, a given $\varphi$ sends $f\in C_c^\infty (G/ K)$ to $\frac{1}{{{\,\textrm{vol}\,}}(K)} f*\varphi$ which lies in $C_c^\infty (G/ K')$ (the volume factor is inserted to be compatible with the “moduli interpretation" below). Similarly, a correspondence between the two sets *G*/*K* and $G/ K'$ also induces a natural map $C_c^\infty (G/ K)\rightarrow C_c^\infty (G/ K')$. In this way we will freely switch between functions and correspondences. We can identify the function $\varphi _{[t, t']}$ in terms of “Hecke correspondences of moduli spaces of vertex lattices", as follows. Let

$$\begin{aligned} \mathbb {N} ^{[t]}=\mathbb {N} _{W_0}^{[t]}=\{\Lambda \subset W_0\mid \Lambda \text { is a vertex lattice of type } t\}. \end{aligned}$$

Note that there is the diagonal action of $\textrm{U}(W_0)$ on $\mathbb {N} ^{[t]}\times \mathbb {N} ^{[t']}$. Then $\mathcal {H} (K_t\backslash \textrm{U} (W_0)/ K_{t'})$ can be identified with the space of functions on $\mathbb {N} ^{[t]}\times \mathbb {N} ^{[t']}$ which are invariant under $\textrm{U}(W_0)$ and have compact support modulo this action.

We also introduce

$$\begin{aligned} \mathbb {N} ^{[t,t']}=\mathbb {N} ^{[t,t']}_{W_0}= \left\{ \begin{array}{ll}\{(\Lambda _t,\Lambda _{t'})\in \mathbb {N} ^{[t]}\times \mathbb {N} ^{[t']}\mid \Lambda _t\subset \Lambda _{t'} \} &  \text {if } t'\le t\\ \{(\Lambda _t,\Lambda _{t'})\in \mathbb {N} ^{[t]}\times \mathbb {N} ^{[t']}\mid \Lambda _{t'}\subset \Lambda _{t} \} &  \text {if } t\le t'. \end{array}\right. \end{aligned}$$

We obtain a diagram,4.1.3or, equivalently, the map

$$\begin{aligned} \pi :\mathbb {N} ^{[t, t']}\rightarrow \mathbb {N} ^{[t]}\times \mathbb {N} ^{[ t']}. \end{aligned}$$

Then

4.1.4 $$\begin{aligned} \varphi _{[t, t']}=\pi _*(\textrm{char}_{\mathbb {N} ^{[t, t']}}). \end{aligned}$$

Note that $\varphi _{[t, t']}$ does not lie in the spherical Hecke algebra. To obtain functions in $\mathcal {H}_K$, we use convolution.

#### Definition 4.1.1

The atomic function associated to an even integer *t* with $0\le t\le n$ is the following element in the spherical Hecke algebra,

4.1.5 $$\begin{aligned} \varphi _{t}={{\,\textrm{vol}\,}}(K_0)^{-1}{{\,\textrm{vol}\,}}(K_t)^{-1}\varphi _{[0,t]}*\varphi _{[t,0]}={{\,\textrm{vol}\,}}(K_t)^{-1}  \textbf{1}_{K_0 K_{t}} *\textbf{1}_{K_t K_{0}}\in \mathcal {H} _K, \end{aligned}$$

where we recall that ${{\,\textrm{vol}\,}}(K_0)=1$.

Consider the composite of correspondences,

$$\begin{aligned} \mathbb {T} _{\le t}=&\mathbb {T} _{W_0, \le t}:=\mathbb {N} ^{[0, t]}\circ \mathbb {N} ^{[t,0]}\\=&\{(\Lambda _0,\Lambda _{t},\Lambda _0')\in \mathbb {N} ^{[0]}\times \mathbb {N} ^{[t]} \times \mathbb {N} ^{[0]} \mid \Lambda _t\subset \Lambda _{0}\cap \Lambda _{0}' \}. \end{aligned}$$

We obtain a diagram with a cartesian square,4.1.6Consider

$$\begin{aligned} \pi : \mathbb {T} _{\le t}\rightarrow \mathbb {N} ^{[0]}\times \mathbb {N} ^{[0]}. \end{aligned}$$

Then, just as in (4.1.4),

4.1.7 $$\begin{aligned} \varphi _{t}=\pi _*(\textrm{char}_{\mathbb {T} _{\le t}}). \end{aligned}$$

#### Remark 4.1.2

As is well-known, the spherical Hecke algebra is commutative. In particular, $\varphi _t*\varphi _{t'}=\varphi _t*\varphi _{t'}$. Comparing the supports of these two functions, we get the equality of the following two subsets of $\mathbb {N} ^{[0]}\times \mathbb {N} ^{[0]}$.

We say that two selfdual lattices $\Lambda _1$ and $\Lambda _2$ are related by a correspondence of type *t*, denoted $\Lambda _1\!\!\iff \!\!\!^t\,\,\Lambda _2$, if there exists a vertex lattice *M* of type *t* contained in $\Lambda _1\cap \Lambda _2$. In other words, $(\Lambda _1, \Lambda _2)\in \pi (\mathbb {T} _{\le t})$. The two sets in question are

4.1.8 $$\begin{aligned} \begin{aligned}&\{(\Lambda _1, \Lambda _2)\mid \exists \Lambda \in \mathbb {N} ^{[0]} \text { with } \Lambda _1\!\!\iff \!\!\!^t\,\,\Lambda \text { and } \Lambda \!\!\iff \!\!\!^{t'}\,\,\Lambda _2\}.\\&\{(\Lambda _1, \Lambda _2)\mid \exists \Lambda \in \mathbb {N} ^{[0]} \text { with } \Lambda _1\!\!\iff \!\!\!^{t'}\,\,\Lambda \text { and } \Lambda \!\!\iff \!\!\!^{t}\,\,\Lambda _2\}. \end{aligned} \end{aligned}$$

Here is an elementary proof of the equality of these two sets that was indicated to us by B. Howard. It is based on Gelfand’s trick of proving the commutativity of the Hecke algebra by constructing an anti-automorphism of $\textrm{U}(W_0)$ inducing the identity on $K\backslash \textrm{U}(W_0)/K$. Fix a basis of $W_0$ such that the hermitian form is given by the antidiagonal unit matrix *J*, and let $\Xi _0$ be the standard lattice. Define the anti-automorphism $\tau$ of $\textrm{U}(W_0)$ by $\tau (g)=J\sigma (g^{-1})J$. Then $\tau$ induces the identity on $A(F_0)$ and hence induces the identity on $\mathcal {H} _K$ (Cartan decomposition). On the other hand, one easily checks that under the identification $K\backslash \textrm{U}(W_0)/K=\textrm{U}(W_0)\backslash (\mathbb {N} ^{[0]}\times \mathbb {N} ^{[0]})$, the map $\tau$ is given by $(\Lambda _0, \Lambda _0')\mapsto (\sigma (\Lambda _0'), \sigma (\Lambda _0))$. It follows that for any $(\Lambda _0, \Lambda _0')\in \mathbb {N} ^{[0]}\times \mathbb {N} ^{[0]}$ there exists $\gamma \in \textrm{U}(W_0)(F_0)$ such that $(\sigma (\Lambda _0'), \sigma (\Lambda _0))=\gamma (\Lambda _0, \Lambda _0')$. Let us now check the claim. By symmetry, it suffices to show that the first of the two sets in (4.1.8) is contained in the second. Let $(\Lambda _1, \Lambda _2)$ be in the first set, i.e., there is $\Lambda \in \mathbb {N} ^{[0]}$ with $\Lambda _1\!\!\iff \!\!\!^t\,\,\Lambda$ and $\Lambda \!\!\iff \!\!\!^{t'}\,\,\Lambda _2$. Let $(\sigma (\Lambda _2), \sigma (\Lambda _1))=\gamma (\Lambda _1, \Lambda _2)$. Then $\sigma (\Lambda _2)\!\!\iff \!\!\!^t\,\,\gamma \Lambda$ and $\gamma \Lambda \!\!\iff \!\!\!^{t'}\,\,\sigma (\Lambda _1)$. It follows that $(\Lambda _1, \Lambda _2)$ is in the second set, with intermediary lattice $\Lambda '=\sigma (\gamma \Lambda )$.

Besides $\mathbb {T} _{\le t}$, we also consider

$$\begin{aligned} \begin{aligned} \mathbb {T} _{t}=\mathbb {T} _{W_0, t}&=\{(\Lambda _0,\Lambda _0')\in \mathbb {N} ^{[0]}\times \mathbb {N} ^{[0]}\mid \Lambda _{0}\cap \Lambda _{0}' \text { is a vertex lattice of type } t\}\\&=\{(\Lambda _0,\Lambda _{t},\Lambda _0')\in \mathbb {N} ^{[0]}\times \mathbb {N} ^{[t]}\times \mathbb {N} ^{[0]}\mid \Lambda _t= \Lambda _{0}\cap \Lambda _{0}' \}, \end{aligned} \end{aligned}$$

with its natural map

$$\begin{aligned} \pi : \mathbb {T} _{ t}\rightarrow \mathbb {N} ^{[0]}\times \mathbb {N} ^{[0]}. \end{aligned}$$

Recall $f^{[t]}:=\textbf{1}_{K\varpi ^{(1^{t/2}, 0^{n-2t}, (-1)^{t/2})}K}$, cf. (3.5.1). Then

4.1.9 $$\begin{aligned} f^{[t]}=\pi _*(\textrm{char}_{\mathbb {T} _{t}}). \end{aligned}$$

#### Proposition 4.1.3

The spherical Hecke algebra $\mathcal {H} _K$ is a polynomial algebra in the atomic functions $\varphi _t$, as *t* runs through all even integers $0\le t\le n$,

$$\begin{aligned} \mathcal {H} _K=\mathbb {Q} [\varphi _2, \varphi _4,\ldots , \varphi _{2m}]. \end{aligned}$$

More precisely, the elements $\{\varphi _{t}\}_{0<t\le n, t\equiv 0\!\!\mod 2}$ are expressed as a linear combination of $\{f^{[t]}\}_{0<t\le n, t\equiv 0\!\!\mod 2}$ by an upper-triangular matrix with all diagonal entries equal to one.

#### Proof

From the “moduli interpretation", we can partition $\mathbb {T} _{\le t}$ into a disjoint union

$$\begin{aligned} \mathbb {T} _{\le t}=\coprod _{t'\le t,\atop t'\equiv 0\!\!\mod 2}\mathbb {T} _{\le t}^{[t']}, \end{aligned}$$

where

$$\begin{aligned} \mathbb {T} _{\le t}^{[t']}=\{(\Lambda _0,\Lambda _{t},\Lambda _0')\in \mathbb {T} _{\le t} \mid \Lambda _{0}\cap \Lambda _{0}' \text { has type } t'\}. \end{aligned}$$

It follows that

4.1.10 $$\begin{aligned} \varphi _t=\sum _{t'\le t,\atop t'\equiv 0\!\!\mod 2 } m(t',t)f^{[t]}, \end{aligned}$$

where $m(t',t)$ is the number of vertex lattices of type *t* contained in a given vertex lattice of type $t'$,

$$\begin{aligned} m(t',t)=\#\{ \Lambda _t\in \mathbb {N} ^{[t]}\mid \Lambda _t\subset \Lambda _{t'}\}. \end{aligned}$$

That is, $m(t',t)$ equals the (constant) degree of the fiber of the natural projection map $\mathbb {N} ^{[t,t']}\rightarrow \mathbb {N} ^{[t']}$. Clearly

$$\begin{aligned} m(t,t)=1. \end{aligned}$$

This shows that the basis $\{\varphi _{t}\}_{0<t\le n, t\equiv 0\!\!\mod 2}$ differs from $\{f^{[t]}\}_{0<t\le n, t\equiv 0\!\!\mod 2}$ by an upper-triangular matrix with all diagonal entries equal to one. This implies the desired assertion. $\square$

#### Remark 4.1.4

One can determine the coefficients $m(t',t)$ explicitly. See [24, Lem. B.2.4] for the formula expressing $\varphi _{2\,m}$ in terms of the $f^{[t]}$.

#### Definition 4.1.5

We call an element $\varphi \in \mathcal {H} _K$ monomial if it can be expressed as a monomial in the atomic functions $\{\varphi _2, \varphi _4,\ldots , \varphi _{2\,m}\}$ (in a necessarily unique way).

It is clear that an arbitrary element $\varphi \in \mathcal {H} _K$ can be expressed as a linear combination of monomial elements (in a unique way).

### The product situation

We also apply the preceding considerations to a product situation. Let $W_0$ be a split space of dimension $n+1$, and let $W_0^\flat =\langle u\rangle ^\perp$, where the special vector $u\in W_0$ has unit length. Then $W_0^\flat \oplus W_0$ is also a split space. We fix a maximal chain of vertex lattices in $W_0$ as in (4.1.1) (where *n* is replaced by $n+1=\dim W_0$)

4.2.1 $$\begin{aligned} \Xi _0\supset \Xi _2\supset \cdots \supset \Xi _{2m}, \end{aligned}$$

and a maximal chain in $W_0^\flat$

4.2.2 $$\begin{aligned} \Xi ^\flat _0\supset \Xi ^\flat _2\supset \cdots \supset \Xi ^\flat _{2m^\flat }, \end{aligned}$$

where $\Xi ^\flat _t$ is a type *t* vertex lattice in $W_0^\flat$, for every even integer $0\le t\le n$. Here $m=\lfloor {(n+1)/2}\rfloor$ and $m^\flat =\lfloor {n/2}\rfloor$. We assume that the self-dual lattice $\Xi _0$ contains the special vector $u_0$, and that $\Xi ^\flat _0$ is related by  Note that we only assume that $u_0\in \Xi _0$, while other lattices $\Xi _t$ ($t>0$) may not contain $u_0$. Then the different choices of $\Xi ^\flat _t$ may yield different parahoric subgroups $K^\flat _t$, but the resulting atomic function $\varphi _t$ is independent of the choices. The Hecke algebra $\mathcal {H} _{K^\flat \times K}$ for $\textrm{U}(W_0^\flat )\times \textrm{U}(W_0)$ can be identified with the tensor product of algebras $\mathcal {H}_{K^\flat }\otimes \mathcal {H}_K$. By an atomic element in $\mathcal {H} _{K^\flat \times K}$ we mean a pure tensor $\widetilde{\varphi }=\varphi ^\flat \otimes \varphi$, where either $\varphi ^\flat$ is atomic and $\varphi$ is the unit element, or $\varphi$ is atomic and $\varphi ^\flat$ is the unit element. A monomial element in $\mathcal {H} _{K^\flat \times K}$ is defined to be a product of atomic elements. This presentation is unique. Any element in $\mathcal {H} _{K^\flat \times K}$ is a linear combination of monomial elements in a unique way.

Let $\varphi _{t, t'}=\varphi _t\otimes \varphi _{t'}$ be an atomic function. Hence either $t=0$ or $t'=0$. Then

4.2.3 $$\begin{aligned} \varphi _{t, t'}=\pi _*( \textrm{char}_{\mathbb {T} _{W_0^\flat ,\le t}\times \mathbb {T} _{W_0,\le t'}}). \end{aligned}$$

Here we use the diagram4.2.4and the corresponding map

$$\begin{aligned} \pi :\mathbb {T} _{W_0^\flat ,\le t}\times \mathbb {T} _{W_0,\le t'}\rightarrow (\mathbb {N} _{W_0^\flat }^{[0]}\times \mathbb {N} _{W_0}^{[0]})\times (\mathbb {N} _{W_0^\flat }^{[0]}\times \mathbb {N} _{W_0}^{[0]}). \end{aligned}$$

The following lemma holds for all functions $\varphi \in C_c^\infty (G_{W_0})$ (and is used earlier in the definition of transfer). We include a proof for the special case of functions in the Hecke algebra, in order to illustrate the idea that will be used in the proof of Proposition 6.1.1.

#### Lemma 4.2.1

Let $\varphi \in \mathcal {H}_{K^\flat }\otimes \mathcal {H}_K$. Then ${{\,\textrm{Orb}\,}}(g,\varphi )$ is finite for every $g\in G_W(F)_{\textrm{rs}}$.

#### Proof

It suffices to consider monomial functions and elements of the form (1, *g*) with $g\in \textrm{U} (W_0)_\textrm{rs}$. We can bound $\varphi$ by a constant multiple of a function of the following form

$$\begin{aligned} \Phi _N=\textbf{1}_{\textrm{U} (W_0^\flat )\cap \varpi ^{-N}{{\,\textrm{End}\,}}_{O_F}(\Xi ^\flat _0)}\otimes \textbf{1}_{\textrm{U} (W_0)\cap \varpi ^{-N}{{\,\textrm{End}\,}}_{O_F}(\Xi _0)} \end{aligned}$$

for some large integer *N*, and hence it suffices to consider such functions $\Phi _N$. In terms of lattice counting, we need to show the finiteness of pairs of self-dual lattices

$$\begin{aligned} (\Lambda ^\flat , \Lambda ), \quad (\Lambda '^\flat , \Lambda ') \end{aligned}$$

such that $\Lambda =\Lambda ^\flat \oplus \langle {u} \rangle , \Lambda '=\Lambda '^\flat \oplus \langle {u} \rangle$, and $\Lambda ^\flat \subset \varpi ^{-N} \Lambda '^\flat$, and $\Lambda \subset \varpi ^{-N} g \Lambda '$. Note that $\Lambda ^\flat$ (resp. $\Lambda '^\flat$) is determined by $\Lambda$ (resp. $\Lambda '$). The self-duality also implies $\Lambda ^\flat \supset \varpi ^{N} \Lambda '^\flat$ and $\Lambda \supset \varpi ^{N} g \Lambda '$.

We claim that $\varpi ^{(2i-2)N}g ^{i-1}u\in \Lambda '$ *and* $\varpi ^{(2i-1)N}g ^iu\in \Lambda$ *for every* $i\ge 1$. To show the claim, it suffices to show

1. $$u\in \Lambda '$$,
2. $$\varpi ^{(2i-2)N}g ^{i-1}u\in \Lambda '\Longrightarrow \varpi ^{(2i-1)N}g ^iu\in \Lambda $$ for every $$i\ge 1$$,
3. $$\varpi ^{(2i-1)N}g ^{i}u\in \Lambda \Longrightarrow \varpi ^{2iN}g ^iu\in \Lambda '$$ for every $$i\ge 1$$.

*Ad *(1): clear.

*Ad* (2): if $\varpi ^{(2i-2)N}g ^{i-1} u\in \Lambda '$ then, from $\Lambda \supset \varpi ^{N} g \Lambda '$, it follows that $\varpi ^{N} g (\varpi ^{(2i-2)N}g ^{i-1} u)=\varpi ^{(2i-1)N}g ^i u\in \Lambda$.

*Ad* (3): if $\varpi ^{(2i-1)N}g ^{i}u\in \Lambda$ then, from $\Lambda ^\flat \subset \varpi ^{-N} \Lambda '^\flat$, it follows that $\Lambda '\supset \varpi ^{N} \Lambda$ and hence $\varpi ^{N}(\varpi ^{(2i-1)N}g ^{i}u)= \varpi ^{2iN} g^i u\in \Lambda '$.

From the claim it follows that $\Lambda$ contains the lattice $\varpi ^{(2n-1)N}\langle {u,gu, \ldots , g^n u} \rangle$, which has full rank by the regular semisimplicity of *g*, cf. [30, Sect. 2.4]. This shows the finiteness of possible $\Lambda$, and hence of $\Lambda '$. This completes the proof. $\square$

## RZ spaces and their Hecke correspondences

In this section we define various Rapoport–Zink spaces (RZ spaces) with certain parahoric levels, and use them to define Hecke correspondences.

### Rapoport–Zink spaces $\mathcal {N}_{n}$ of self-dual level

Let *S* be a ${{\,\textrm{Spf}\,}}{O_{\breve{F}}}$-scheme. Consider a triple $(X, \iota ,\lambda )$ where

(i) *X* is a formal $\varpi$-divisible $O_{F_0}$-module over *S* of relative height 2*n* and dimension *n*,
(ii) $\iota : O_F\rightarrow {{\,\textrm{End}\,}}(X)$ is an action of $O_F$ extending the $O_{F_0}$-action and satisfying the Kottwitz condition of signature $(1,n-1)$: for all $a\in O_F$, the characteristic polynomial of $\iota (a)$ on ${{\,\textrm{Lie}\,}}X$ is equal to $(T-a)(T-\sigma (a))^{n-1}\in \mathcal {O}_S[T]$,
(iii) $\lambda : X\rightarrow X^\vee$ is a principal polarization on *X* whose Rosati involution induces the automorphism $\sigma$ on $O_F$ via $\iota$.

Up to $O_F$-linear quasi-isogeny compatible with polarizations, there is a unique such basic triple $(\mathbb {X}, \iota _{\mathbb {X}}, \lambda _{\mathbb {X}})$ over $S={{\,\textrm{Spec}\,}}{\bar{k}}$. Let $\mathcal {N}_{n}=\mathcal {N}_{F/F_0, n}$ be the (relative) *unitary Rapoport–Zink space of self-dual level*, which is a formal scheme over ${{\,\textrm{Spf}\,}}{O_{\breve{F}}}$ representing the functor sending each *S* to the set of isomorphism classes of tuples $(X, \iota , \lambda , \rho )$, where the *framing* $\rho : X\times _S {\bar{S}}\rightarrow \mathbb {X}\times _{{{\,\textrm{Spec}\,}}{\bar{k}}}{\bar{S}}$ is an $O_F$-linear quasi-isogeny of height 0 such that $\rho ^*((\lambda _\mathbb {X})_{{\bar{S}}})=\lambda _{{\bar{S}}}$. Here ${\bar{S}}{:=}S_{{\bar{k}}}$ is the special fiber.

The Rapoport–Zink space $\mathcal {N}_{n}$ is formally locally of finite type and formally smooth of relative dimension $n-1$ over ${{\,\textrm{Spf}\,}}{O_{\breve{F}}}$ ( [32, 25, Prop. 1.3]).

### The hermitian space $\mathbb {V}_n$

Let $n\ge 1$ be an integer. Let $\mathbb {E}$ be the formal $O_{F_0}$-module of relative height 2 and dimension 1 over ${{\,\textrm{Spec}\,}}{\bar{k}}$. Then $D{:=}{{\,\textrm{End}\,}}_{O_{F_0}}^\circ (\mathbb {E}):={{\,\textrm{End}\,}}_{O_{F_0}}(\mathbb {E}) \otimes \mathbb {Q}$ is the quaternion division algebra over $F_0$. We fix an $F_0$-embedding $\iota _\mathbb {E}:F\rightarrow D$, which makes $\mathbb {E}$ into a formal $O_F$-module of relative height 1. We fix an $O_{F_0}$-linear principal polarization $\lambda _\mathbb {E}: \mathbb {E}\xrightarrow {\sim } \mathbb {E}^\vee$. Then $(\mathbb {E}, \iota _\mathbb {E},\lambda _\mathbb {E})$ is a hermitian $O_F$-module of signature (1, 0). We have $\mathcal {N}_1\simeq {{\,\textrm{Spf}\,}}{O_{\breve{F}}}$ and there is a unique lifting (*the canonical lifting*) $\mathcal {E}$ of the formal $O_F$-module $\mathbb {E}$ over ${{\,\textrm{Spf}\,}}{O_{\breve{F}}}$, equipped with its $O_F$-action $\iota _\mathcal {E}$, its framing $\rho _\mathcal {E}: \mathcal {E}_{{\bar{k}}}\xrightarrow {\sim }\mathbb {E}$, and its principal polarization $\lambda _\mathcal {E}$ lifting $\rho _\mathcal {E}^*(\lambda _\mathbb {E})$. Define ${\overline{\mathbb {E}}}$ to be the same $O_{F_0}$-module as $\mathbb {E}$ but with $O_F$-action given by $\iota _{{\overline{\mathbb {E}}}}{:=}\iota _\mathbb {E}\circ \sigma$, and $\lambda _{{\overline{\mathbb {E}}}}{:=}\lambda _{\mathbb {E}}$, and similarly define $\bar{\mathcal {E}}$ and $\lambda _{\bar{\mathcal {E}}}$.

Denote by $\mathbb {V}=\mathbb {V}_n:={{\,\textrm{Hom}\,}}_{O_F}^\circ ({\overline{\mathbb {E}}}, \mathbb {X})$ the space of special quasi-homomorphisms. Then $\mathbb {V}$ carries a $F/F_0$-hermitian form: for $x,y\in \mathbb {V}$, the pairing $(x,y)\in F$ is given by the composition

$$\begin{aligned} ({\overline{\mathbb {E}}}\xrightarrow {x} \mathbb {X}\xrightarrow {\lambda _\mathbb {X}} {\mathbb {X}^\vee }\xrightarrow {y^\vee } {\overline{\mathbb {E}}}^\vee \xrightarrow {\lambda _\mathbb {E}^{-1}}{\overline{\mathbb {E}}})\in {{\,\textrm{End}\,}}_{O_{F}}^\circ ({\overline{\mathbb {E}}})=\iota _{\overline{\mathbb {E}}}(F)\simeq F. \end{aligned}$$

The hermitian space $\mathbb {V}$ is the unique (up to isomorphism) non-degenerate non-split $F/F_0$-hermitian space of dimension *n*. The unitary group $\textrm{U}(\mathbb {V})(F_0)$ acts on the framing hermitian $O_F$-module $(\mathbb {X}, \iota _\mathbb {X},\lambda _\mathbb {X})$ (via the identification in [17, Lem. 3.9]) and hence acts on the Rapoport–Zink space $\mathcal {N}_n$ via $g(X,\iota ,\lambda ,\rho )=(X, \iota ,\lambda , g\circ \rho )$ for $g\in \textrm{U}(\mathbb {V})(F_0)$.

To a non-zero $u\in \mathbb {V}$, there is the associated special divisor $\mathcal {Z} (u)$ on $\mathcal {N} _n$. It is the closed formal subscheme of $\mathcal {N} _n$ of points $(X, \iota , \lambda , \rho )$ where the quasi-homomorphism $u: \mathbb {E} \rightarrow \mathbb {X}$ lifts to a homomorphism $\mathcal {E} \rightarrow X$, cf. [17]. More generally, for any finitely generated subgroup $L\subset \mathbb {V}$, there is the associated *special cycle* $\mathcal {Z} (L)$, the locus where the quasi-homomorphisms $u: \mathbb {E} \rightarrow \mathbb {X}$ lift to homomorphisms $\mathcal {E} \rightarrow X$, for all $u\in L$.

### RZ spaces $\mathcal {N} _n^{[t]}$

Let *t* be an even integer with $0\le t\le n$. Now we consider triples $(Y, \iota _Y,\lambda _Y)$ as in Sect. 5.1 (with *Y* instead of *X*), except that we replace the condition on the polarization to be principal by the condition that the polarization is of degree $q^{2t}$ and satisfies $\ker (\lambda _Y)\subset Y[\varpi ]$. Again, we fix a framing object $(\mathbb {X} _t, \iota _{\mathbb {X} _t}, \lambda _{\mathbb {X} _t})$ over ${{\,\textrm{Spec}\,}}{\bar{k}}$ (which is again unique up to $O_F$-linear quasi-isogeny compatible with polarizations). We define the RZ-space $\mathcal {N} _n^{[t]}$ as the space of tuples $(Y, \iota _Y,\lambda _Y, \rho _Y)$, where $\rho _Y$ is a framing with $(\mathbb {X} _t, \iota _{\mathbb {X} _t}, \lambda _{\mathbb {X} _t})$. It is an RZ space of level equal to the stabilizer of a vertex lattice of type *t*. Note that $\mathcal {N} ^{[0]}_n=\mathcal {N} _n$. The Rapoport–Zink space $\mathcal {N}_{n}^{[t]}$ is formally locally of finite type and regular of dimension *n* with semi-stable reduction over ${{\,\textrm{Spf}\,}}{O_{\breve{F}}}$ [12, 32].

### RZ spaces $\mathcal {N} _n^{[t,t']}$

Let $t, t'$ be even integers. We introduce the RZ-space $\mathcal {N} _n^{[t, t']}$. Let first $t'\le t$. We fix a $O_{F_0}$-linear isogeny of degree $q^{t-t'}$ with kernel killed by $\varpi$,

5.4.1 $$\begin{aligned} \varphi _{t, t'}:\mathbb {X} _{t}\rightarrow \mathbb {X} _{t'}, \end{aligned}$$

such that $\varphi ^*(\lambda _{\mathbb {X} _{t'}})=\lambda _{\mathbb {X} _t}$. Then $\mathcal {N} _n^{[t, t']}$ classifies triples

$$\begin{aligned} \big ((X_1, \iota _1,\lambda _1, \rho _1), (X_2, \iota _2,\lambda _2, \rho _2), \varphi :X_1\rightarrow X_2\big ), \end{aligned}$$

where $(X_1, \iota _1,\lambda _1, \rho _1)\in \mathcal {N} ^{[t]}$ and $(X_2, \iota _2,\lambda _2, \rho _2)\in \mathcal {N} ^{[t']}$, and where $\varphi$ is an isogeny lifting $\varphi _{t, t'}:\mathbb {X} _{t}\rightarrow \mathbb {X} _{t'}$. Then $\varphi$ is uniquely determined, is of degree $q^{t-t'}$ and satisfies $\ker \varphi \subset X_1[\varpi ]$; also, $\varphi$ preserves the polarizations.

When $t\le t'$, we define $\mathcal {N} _n^{[t, t']}$ to be the *transpose* $^t\mathcal {N} _n^{[t', t]}$ of $\mathcal {N} _n^{[t', t]}$, i.e., $\mathcal {N} _n^{[t, t']}$ classifies triples

$$\begin{aligned} \big ((X_1, \iota _1,\lambda _1, \rho _1), (X_2, \iota _2,\lambda _2, \rho _2), \varphi :X_2\rightarrow X_1\big ), \end{aligned}$$

where $(X_1, \iota _1,\lambda _1, \rho _1)\in \mathcal {N} ^{[t]}$ and $(X_2, \iota _2,\lambda _2, \rho _2)\in \mathcal {N} ^{[t']}$, and where $\varphi$ is an isogeny lifting $\varphi _{t', t}:\mathbb {X} _{t'}\rightarrow \mathbb {X} _{t}$. Only the cases $(t,t')=(0,t')$ and $(t,t')=(t,0)$ will be effectively used in this paper. We view $\mathcal {N} _n^{[t,t']}$ as a correspondence via the two natural projections, which is analogous to the corresponding diagram (4.1.3) of lattice correspondences,5.4.2The two projection maps are both proper (representable by a projective morphism). Note that $\mathcal {N} _n^{[t,t']}$ is an RZ space of level equal to the joint stabilizer of two vertex lattices of type $t, t'$. It is formally locally of finite type and regular of dimension *n* with semi-stable reduction over ${{\,\textrm{Spf}\,}}{O_{\breve{F}}}$ [12, 32]).

### The Hecke correspondence $\mathcal {T} _n^{\le t}$

Define $$\mathcal {T} _n^{\le t}$$ by

$$\begin{aligned} \big ((Y, \iota _Y,\lambda _Y, \rho _Y),(X_1, \iota _1,\lambda _1, \rho _1), (X_2, \iota _2,\lambda _2, \rho _2), \varphi _i: Y\rightarrow X_i, i=1,2\big ) \end{aligned}$$

such that $(Y,X_i,\varphi _i)\in \mathcal {N} _n^{[t,0]}$. In other words, $\mathcal {T} _n^{\le t}$ is the composition of correspondences,

$$\begin{aligned} \mathcal {T} _n^{\le t}= \mathcal {N} _n^{[0,t]}\circ \mathcal {N} _n^{[t,0]}, \end{aligned}$$

cf. Sect. 9.2. Explicitly, this means that we obtain the following diagram with a cartesian square in the middle,5.5.1Note that all formal schemes in the lower two rows are regular and the maps are relatively representable by morphisms of projective schemes. Using the calculus in the “Appendix”, we may therefore define for closed formal subschemes $A\subset \mathcal {N} _n^{[0]}$, resp. $B\subset \mathcal {N} ^{[t]}_n$

5.5.2 $$\begin{aligned} \begin{aligned}&\mathbb {T} _t^{A, +}=(\mathcal {N} ^{[0,t]}_n)_*:K^A(\mathcal {N} ^{[0]}_n)\rightarrow K^{\mathcal {N} ^{[0,t]}_n(A)}(\mathcal {N} ^{[t]}_n), \\&\mathbb {T} _t^{B, -}=(\mathcal {N} ^{[t, 0]}_n)_*:K^B(\mathcal {N} ^{[t]}_n)\rightarrow K^{\mathcal {N} ^{[t, 0]}_n(B)}(\mathcal {N} ^{[0]}_n). \end{aligned} \end{aligned}$$

This defines the map

5.5.3 $$\begin{aligned} \mathbb {T} ^A_t= \mathbb {T} ^{\mathcal {N} ^{[0,t]}_n(A), -}_t\circ \mathbb {T} ^{A, +}_t:K^A(\mathcal {N} _n)\rightarrow K^{\mathcal {T} ^{\le t}(A)}(\mathcal {N} _n). \end{aligned}$$

Recall here that $\mathcal {N} _n=\mathcal {N} _n^{[0]}$.

We state the following conjecture. Note that this conjecture is empty for $n=2$ and $n=3$.

#### Conjecture 5.5.1

Let $t\ne t'$. Then for any closed formal subscheme *A* of $\mathcal {N} _n$ we have an equality of the two closed formal subschemes $\mathcal {T} ^{\le t}(\mathcal {T} ^{\le t'}(A))$ and $\mathcal {T} ^{\le t'}(\mathcal {T} ^{\le t}(A))$ of $\mathcal {N} _n$. Furthermore, the two maps

$$\begin{aligned}  &   \mathbb {T} ^{\mathcal {T} ^{\le t'}(A)}_t\circ \mathbb {T} ^A_{t'}:K^A(\mathcal {N} _n)\rightarrow K^{\mathcal {T} ^{\le t}(\mathcal {T} ^{\le t'}(A))}(\mathcal {N} _n), \quad \\  &   \mathbb {T} ^{\mathcal {T} ^{\le t}(A)}_{t'}\circ \mathbb {T} ^A_{t}:K^A(\mathcal {N} _n)\rightarrow K^{\mathcal {T} ^{\le t'}(\mathcal {T} _{t}(A))}(\mathcal {N} _n) \end{aligned}$$

are identical modulo torsion.

#### Remark 5.5.2

The conjectured equality $\mathcal {T} ^{\le t}(\mathcal {T} ^{\le t'}(A))=\mathcal {T} ^{\le t'}(\mathcal {T} ^{\le t}(A))$ is analogous to the identity of the two subsets (4.1.8) of $\mathbb {N} ^{[0]}\times \mathbb {N} ^{[0]}$ in Remark 4.1.2.

For an atomic function $\varphi _t$ (cf. Definition 4.1.1) with $0\le t\le n$, we define the corresponding Hecke operator as

$$\begin{aligned} \mathbb {T} ^A_{\varphi _t}:=\mathbb {T} ^A_t:K^A(\mathcal {N} _n)\rightarrow K^{\mathcal {T} ^{\le t}(A)}(\mathcal {N} _n). \end{aligned}$$

The homomorphisms obtained in this way will be called *atomic Hecke operators*. By Proposition 4.1.3, atomic functions form a basis of the spherical Hecke algebra (as a polynomial algebra). Following [11, Lem. 1.4], we introduce

$$\begin{aligned} K^\sigma (\mathcal {N})_\mathbb {Q} =\oplus _{A\subset \mathcal {N} _n}\, K^A(\mathcal {N} _n)\otimes \mathbb {Q}, \end{aligned}$$

where *A* runs through all closed formal subschemes defined by radical ideal sheaves. Then we obtain for every *t* an endomorphism $\mathbb {T} _{\varphi _t}$ of $K^\sigma (\mathcal {N} _n)_\mathbb {Q}$ by mapping an element $(\gamma _A)_A$ to $(\beta _B)_B$, where

$$\begin{aligned} \beta _B=\sum _{\{A\mid \mathcal {T} ^{\le t}(A)\equiv B\}}\gamma _A. \end{aligned}$$

Here, the notation means that the radical of the ideal defining the closed formal subscheme $\mathcal {T} ^{\le t}(A)$ coincides with the ideal sheaf of *B*.

Denoting by $\widetilde{\mathcal {H}}_K$ the non-commutative polynomial algebra over $\mathbb {Q}$ in the indeterminates $X_1,\ldots ,X_m$, we therefore obtain by taking compositions a homomorphism of algebras

5.5.4 $$\begin{aligned} \mathbb {T} :\widetilde{\mathcal {H}}_K\rightarrow {{\,\textrm{End}\,}}(K^\sigma (\mathcal {N} _n)_\mathbb {Q}). \end{aligned}$$

Explicitly, if $\varphi =\varphi _1*\varphi _2*\cdots *\varphi _r$ is a monomial in the atomic Hecke functions $\varphi _1=\varphi _{t_1}, \varphi _2=\varphi _{t_2},\ldots ,\varphi _r=\varphi _{t_r}$, then $\mathbb {T} ^A_\varphi ={\mathbb {T} ^{\mathcal {T} _{\le t_{2}}\circ \mathcal {T} _{\le t_{3}}\circ \ldots \circ \mathcal {T} _{\le t_{r}}(A) }_{t_1}}\circ \cdots \circ {\mathbb {T} _{\le t_{r-1}}^{\mathcal {T} _{\le t_r}(A)}}\circ {\mathbb {T} _{\le t_r}^A}$. These endomorphisms of $K^\sigma (\mathcal {N} _n)_\mathbb {Q}$ will be called *monomial* Hecke operators.

Conjecture 5.5.1 implies that this homomorphism factors through a homomorphism of the Hecke algebra,

5.5.5 $$\begin{aligned} \mathbb {T} :\mathcal {H} _K\rightarrow {{\,\textrm{End}\,}}(K^\sigma (\mathcal {N} _n)_\mathbb {Q}). \end{aligned}$$

#### Remark 5.5.3

Let $n>3$. Then in general

$$\begin{aligned} (\mathcal {T} _{\le t_1}\circ \mathcal {T} _{\le t_2}\circ \ldots \circ \mathcal {T} _{\le t_r})_*\ne (\mathcal {T} _{\le t_1})_*\circ (\mathcal {T} _{\le t_2})_*\circ \ldots \circ (\mathcal {T} _{\le t_r})_*. \end{aligned}$$

In other words, the composed correspondence (defined by iterated fiber products) does not induce the composition of induced correspondences. This is why we do not define $\mathbb {T} _{\varphi _1*\varphi _2*\cdots *\varphi _r}$ as the map induced by the composed geometric correspondence $\mathcal {T} ^{\le t_1}\circ \mathcal {T} ^{\le t_2}\circ \cdots \circ \mathcal {T} ^{\le t_r}$. To see the difficulty, consider the *r*-fold iterate $\mathbb {T} _t^r$ for some $t>0$ and for variable *r*. By [13, Thm. 1.2], the fiber dimension of the map $\mathcal {N} _n^{[0,t]}\rightarrow \mathcal {N} _n^{[0]}$ is positive, and hence so is the fiber dimension *d* of $\mathcal {T} _n^{\le t}\rightarrow \mathcal {N} _n^{[0]}$. But then the fiber dimension of the *r*-fold composition of geometric correspondences $\mathcal {T} ^{\le t}\circ \mathcal {T} ^{\le t}\circ \cdots \circ \mathcal {T} ^{\le t}\rightarrow \mathcal {N} _n^{[0]}$ is equal to *rd* and hence, for $rd>n$, has irreducible components contained in the special fiber which have dimension larger than the dimension of the generic fiber. Hence the resulting formal scheme is not flat over $O_{\breve{F}}$ and the induced map on K-groups is not equal to the *r*-fold composition of the endomorphism $(\mathcal {T} ^{\le t})_*$.

This problem would disappear, if we had defined the Hecke correspondences as derived formal schemes. Indeed, the map $\mathbb {T} _{\varphi _1*\varphi _2*\cdots *\varphi _r}$ is induced by the composition of derived geometric correspondences $\mathcal {T} _{\textrm{der}}^{\le t_1}\circ \mathcal {T} _{\textrm{der}}^{\le t_2}\circ \cdots \circ \mathcal {T} _{\textrm{der}}^{\le t_r}$, cf. Remark 9.2.2. As the argument above shows, this composition of derived geometric correspondences is not in general a classical formal scheme in our case. Note that, even if we had defined our Hecke operators in terms of derived formal schemes, this does not seem to help in proving Conjecture 5.5.1. Indeed, the only potential argument we see to prove this commutativity is to relate our Hecke operators to classical Hecke operators in the generic fiber, where the desired commutativity holds, and use some density argument to extend the commutativity integrally. However, derived schemes seem unsuitable for such density arguments.

#### Remark 5.5.4

It is conceivable that $(\mathcal {T} ^{\le t})_*=\mathbb {T} _t$, even when the fiber dimension is positive, i.e., when the maps from $\mathcal {T} ^{\le t}$ to $\mathcal {N} _n$ are not flat. Even so, we prefer to write $\mathbb {T} _t$ as a product of intertwining Hecke operators. Our definition is tentative and only a proof of the AFL for the full Hecke algebra in higher dimension will decide which definition is “the right one”.

#### Remark 5.5.5

The argument above uses the fiber dimension of the natural maps $\pi :\mathcal {N} _n^{[t, 0]}\rightarrow \mathcal {N} _n^{[0]}$, resp. $\pi ':\mathcal {N} _n^{[0, t]}\rightarrow \mathcal {N} _n^{[0]}$. In [13], the natural maps $\pi :\mathcal {N} _n^{[t', t]}\rightarrow \mathcal {N} _n^{[t']}$ for arbitrary $t'\ne t$ are considered (in loc. cit. arbitrary affine Deligne-Lusztig varieties are considered at the point set level). More precisely, consider the fiber of $\pi$ over an arbitrary point in $\mathcal {N} _n^{[t']}({\bar{k}})$ (this fiber is the set of ${\bar{k}}$-points of a projective scheme over ${\bar{k}}$). Then, for $n>2$, such a fiber always has strictly positive dimension, unless $t=0$ and $t'=2$, in which case the fibers are finite, cf. [13, Thm. 1.2]. Hence the morphism $\pi$ is non-flat in all cases outside the case $(t=0, t'=2)$. Indeed, both morphisms $\pi$ and $\pi '$ are relatively representable by projective morphisms which are finite in the generic fiber. If flatness held, then all fibers in the special fiber would be finite as well, a contradiction.

#### Example 5.5.6

Let $n=2$. In this case, $\mathcal {N} _2$ can be identified with the Lubin-Tate space $\mathcal {M} _2$ for $n=2$ via the Serre construction, cf. [17]. Here $\mathcal {M} _2$ parametrizes triples $(Y, \rho )$, where *Y* is a strict formal $O_{F_0}$-module of dimension one and height 2, and where $\rho$ is a framing with a fixed Lubin-Tate module $\mathbb {Y}$ over ${\bar{k}}$. Also, there is a natural identification of $\mathcal {N} _2^{[2]}$ with $\mathcal {N} _2$. Indeed, let $(Y, \iota _Y,\lambda _Y, \rho _Y)$ be an object of $\mathcal {N} _2^{[2]}$. Then the inclusion $\ker \lambda _Y\subset Y[\varpi ]$ is an equality and hence $\lambda _Y=\varpi \lambda$ for a unique principal polarization $\lambda$; associating to $(Y, \iota _Y,\lambda _Y, \rho _Y)$ the object $(Y, \iota _Y,\lambda , \rho _Y)$ of $\mathcal {N} _2$ defines the desired isomorphism. Under this identification, $\mathcal {N} _2^{[2,0]}$ is isomorphic to $\mathcal {M} _{2, \Gamma _0}$, the $\Gamma _0(p)$-level covering of $\mathcal {M} _2$ (generalized from $\mathbb {Q} _p$ to arbitrary $F_0$). Here $\mathcal {M} _{0, \Gamma _0}$ parametrizes isogenies $\alpha : Y\rightarrow Y'$ of degree *q* with $\ker \alpha \subset Y[\varpi ]$ which lift a given isogeny $\mathbb {Y} \rightarrow \mathbb {Y}$. Note that $\mathcal {M} _{2, \Gamma _0}({\bar{k}})$ consists of a single point. Indeed, any two isogenies $\mathbb {Y} \rightarrow \mathbb {Y}$ as above differ by a quasi-isogeny of degree 0. But such a quasi-isogeny is automatically an automorphism: denoting by *D* the quaternion division algebra over $F_0$, we have

$$\begin{aligned} {{\,\textrm{Aut}\,}}(\mathbb {Y})=O_D^\times =\{x\in D\mid {{\,\textrm{Nm}\,}}(x)\in O_{F_0}^\times \}=\{x\in {{\,\textrm{End}\,}}^o(\mathbb {Y})\mid \deg (x)=0\}. \end{aligned}$$

In this case, all maps to $\mathcal {N} _2$ in the diagram (5.5.1) are finite and flat, and hence so are the corresponding maps for the iterated correspondences $(\mathcal {T} ^{\le 2})^r=\mathcal {T} ^{\le 2}\circ \mathcal {T} ^{\le 2}\circ \cdots \circ \mathcal {T} ^{\le 2}$. In this case, Conjecture 5.5.1 is empty, we have $(\mathcal {T} _{\le 2})_* =(\mathcal {N} _2^{[2,0]})_*\circ (\mathcal {N} _2^{[0,2]})_*$ and the Hecke operators are induced by geometric Hecke correspondences, i.e., $\mathbb {T} _t^r=((\mathcal {T} ^{\le 2})^r)_*$, cf. Lemma 9.2.1.

#### Remark 5.5.7

Li–Mihatsch [23] consider a situation related to the Linear ATC/AFL for Lubin-Tate space. They also define Hecke operators by first constructing these for distinguished generators of the Hecke algebra, and then by composition (instead of maps of K-groups, they consider maps of cycle groups). In their case both projection maps to $\mathcal {N} _n$ are finite and flat, and the same is true for the composition of these distinguished geometric correspondences. Hence their compositions of distinguished Hecke operators are induced by compositions of geometric correspondences. In their case, the analogue of Conjecture 5.5.1 follows by a density argument for the Hecke correspondences from the classical definition of Hecke correspondences in the generic fiber, which implies the commutativity in the generic fiber.

#### Remark 5.5.8

Fix an even *t* with $0\le t\le n$. Since $\mathcal {N} _n^{[t']}$ is regular for all $t'$, the diagram (5.4.2) defines a Hecke operator $\mathbb {T} _{\textbf{1}_{K^{[t]}K^{[t, t']}}*\textbf{1}_{K^{[t',t]}K^{[t]}}}$ corresponding to the element $\textbf{1}_{K^{[t]}K^{[t, t']}}*\textbf{1}_{K^{[t', t]}K^{[t]}}$ in the Hecke algebra $\mathcal {H} _{K^{[t]}}$. When $t>0$, it is not clear what subalgebra these elements generate. It is also not clear what the relations are among these elements.

### Hecke correspondences for the product

Let

$$\begin{aligned} \mathcal {N} _{n,n+1}= \mathcal {N}_{n}\times _{{{\,\textrm{Spf}\,}}O_{\breve{F}}}\mathcal {N}_{n+1}. \end{aligned}$$

We replace the diagram (5.4.2) by the following diagram, in which the top is defined by the fact that the square is cartesian,We use this diagram to define the Hecke operator $\mathbb {T} _\varphi$ when $\varphi \in \mathcal {H} _{K^\flat \times K}$ is atomic. In this case $t=0$ or $t'=0$ and hence the lower oblique arrows are the identity in one factor; we define $\mathbb {T} _\varphi =\mathbb {T} _\varphi ^-\circ \mathbb {T} _\varphi ^+$ as in (5.5.3). More precisely, for closed formal subschemes *A* of $\mathcal {N} _{n, n+1}$, resp. *B* of $\mathcal {N} _n^{[t]}\times \mathcal {N} _{n+1}^{[t']}$, we obtain

5.6.1 $$\begin{aligned} \begin{aligned}&\mathbb {T} ^{A, +}_\varphi :K^A(\mathcal {N} _{n, n+1}) \rightarrow K^{(\mathcal {N} _n^{[0,t]}\times \mathcal {N} _{n+1}^{[0,t']})(A)}( \mathcal {N} _n^{[t]}\times \mathcal {N} _{n+1}^{[t']}), \\&\mathbb {T} ^{B, -}_\varphi :K^B( \mathcal {N} _n^{[t]}\times \mathcal {N} _{n+1}^{[t']})\rightarrow K^{(\mathcal {N} _n^{[t, 0]}\times \mathcal {N} _{n+1}^{[t', 0]})(B)}(\mathcal {N} _{n, n+1}),\\&\mathbb {T} ^A_\varphi :K^A(\mathcal {N} _{n, n+1})\rightarrow K^{\mathcal {T} ^{\le t, \le t'}(A)}( \mathcal {N} _{n, n+1}). \end{aligned} \end{aligned}$$

After this, assuming the obvious analogue of Conjecture 5.5.1 for $\mathcal {N} _{n, n+1}$, we define $\mathbb {T} ^A_\varphi$ for monomial elements by iterated compositions, and for general elements $\varphi \in \mathcal {H} _{K^\flat \times K}$ as linear combinations.

## AFL conjectures for the Hecke algebra

In this section, we formulate the AFL conjecture in its homogeneous version and in its inhomogeneous version. We consider the arithmetic diagonal cycle $\Delta \subseteq \mathcal {N} _{n,n+1}$.

### Statement of the AFL for Hecke correspondences (homogeneous version)

Let $W_0$ be a split hermitian space of dimension $n+1$ and $W_1$ its nonsplit form. Also, let $u_i\in W_i$ of unit norm for $i=0, 1$. We identify $W_1$ with $\mathbb {V}_{n+1}$ defined in Sect. 5.2 in such a way that $u_1\in W_1$ is mapped to the element $u_0\in \mathbb {V} _{n+1}$ which corresponds to the map ${\bar{\mathbb {E}}}\rightarrow \mathbb {X}$ defined by the product decomposition $\mathbb {X} =\mathbb {X} ^\flat \times {\bar{\mathbb {E}}}$. Then we may identify $\textrm{U}(W_1^\flat )$ (resp. $\textrm{U}(W_1)$) with $\textrm{U}(\mathbb {V}_{n})$ (resp. $\textrm{U}(\mathbb {V}_{n+1})$). Then $G_{W_1}(F_0)$ acts on $\mathcal {N}_{n}\times \mathcal {N}_{n+1}$ via this identification.

For each monomial element $\varphi \in \mathcal {H}_{K^\flat }\otimes \mathcal {H}_K$ (cf. Definition 4.1.5), we consider the corresponding geometric correspondence $\mathcal {T} _\varphi$ with its two projections $\pi _1, \pi _2:\mathcal {T} _\varphi \rightarrow \mathcal {N} _{n, n+1}$ (recall that in general the Hecke operator corresponding to $\varphi$ is not induced by $\mathcal {T} _\varphi$). Note that $\mathcal {T} _\varphi$ depends on the order of the product decomposition of $\varphi$ into atomic elements. Let $g\in G_{W_1}(F_0)$, and consider the image $g\Delta$ under the induced automorphism of $\mathcal {N} _{n, n+1}$.

#### Proposition 6.1.1

Let $\varphi \in \mathcal {H}_{K^\flat }\otimes \mathcal {H}_K$ be a monomial element. Let $g\in G_{W_1}(F_0)_\textrm{rs}$ be regular semisimple. Then the intersection

$$\begin{aligned} g\Delta \cap \mathcal {T} _\varphi ( \Delta )=\pi _2\big (\pi _2^{-1}(g\Delta )\times _{\mathcal {T} _\varphi } \pi _1^{-1}(\Delta )\big ) \end{aligned}$$

is a proper scheme, i.e., its ideal of definition contains an ideal of definition of $\mathcal {N} _{n, n+1}$, and the underlying reduced scheme is proper over ${{\,\textrm{Spec}\,}}{\bar{k}}$.

#### Proof

The proof is based on the same idea as that of Lemma 4.2.1. It suffices to consider elements of the form (1, *g*) with $g\in \textrm{U} (W_1)_\textrm{rs}$. Introduce the locus $\mathcal {T} _N$ of points $\big ((X^\flat , X), (X^{\prime \flat }, X')\big )$ in $\mathcal {N} _{n, n+1}\times \mathcal {N} _{n, n+1}$ such that the universal quasi-isogenies $f^\flat :X^\flat \rightarrow X^{\prime \flat }$ and $f:X\rightarrow X'$ defined by the framings, are isogenies after being multiplied by $\varpi ^N$ (here we have suppressed all auxiliary structures from the notation). It is well-known that $\mathcal {T}_N$ is a closed formal subscheme, cf. [32, Sect. 2]. Then there exists *N* such that ${{\,\textrm{im}\,}}(\mathcal {T} _\varphi )\subset \mathcal {T} _N$. It suffices to prove that $(\Delta \times (1\times g)\Delta )\cap \mathcal {T} _N$ is a proper scheme.

The two projection maps from $\mathcal {T} _N$ to $\mathcal {N} _{n, n+1}$ are both proper. Therefore it suffices to show that the image of the intersection under the first projection map is a proper scheme. Let $(X^\flat ,X)$, resp. $(X'^\flat ,X')$, be points in $\Delta$ such that $\big ((X^\flat ,X), (X'^\flat ,g X')\big )$ lies in $(\Delta \times (1\times g)\Delta )\cap \mathcal {T} _N$. Then $X=X^\flat \times \mathcal {E}$ and $X'=X'^\flat \times \mathcal {E}$, and the natural quasi-isogenies $f^\flat _1:X^\flat \rightarrow X'^\flat$ and $f_2:X\rightarrow g X'$ have the property that $\varpi ^N f_1^\flat , \varpi ^N f_2$ are isogenies. Consider the quasi-isogeny $f_1=(f_1^\flat ,\textrm{id} _{\mathcal {E}}): X\rightarrow X'$. Then $\varpi ^N f_1$ is an isogeny.

Recall the element $u_0\in \mathbb {V}$ corresponding to $u_1\in W_1$, i.e., the natural element corresponding to the product decomposition $\mathbb {X} =\mathbb {X} ^\flat \times {\bar{\mathbb {E}}}$. Let $\mathcal {Z} (u_0)$ be the associated special divisor on $\mathcal {N} _{n+1}$, cf. Sect. 5.2. From $X'\in \mathcal {Z} (u_0)$ and the isogeny $\varpi ^N f_2$, it follows that $X\in \mathcal {Z} (\varpi ^N gu_0)$. From the isogeny $\varpi ^N f_1$ it follows that $X'\in \mathcal {Z} (\varpi ^{2N} gu_0)$. Then inductively we see that *X* lies on the intersection of the special divisors $\mathcal {Z} (u_0), \mathcal {Z} (\varpi ^N gu_0), \mathcal {Z} (\varpi ^{3N} g^2u_0),\ldots$. In particular, *X* lies on the special cycle

$$\begin{aligned} \mathcal {Z} (\varpi ^{(2n-1)N}\langle { u_0, gu_0, \ldots , g^n u_0} \rangle ). \end{aligned}$$

It is known that this special cycle is a proper scheme, cf. [25, Proof of Lem. 6.1]. This shows that the image $(X^\flat ,X)$ of the intersection under the first projection map is a proper scheme. The proof is complete. $\square$

We introduce the *intersection number*

6.1.1 $$\begin{aligned} {{\,\mathrm{\textrm{Int}}\,}}(g,\varphi ):=\langle g\Delta , \mathbb {T} ^\Delta _\varphi (\Delta ) \rangle _{\mathcal {N} _{n,n+1}}, \quad g\in G_{W_1}(F_0)_\textrm{rs}, \quad \varphi \in \mathcal {H} _{K^\flat \times K}. \end{aligned}$$

Here the RHS is defined by linearity from the case of monomial $\varphi$. For monomial $\varphi$, it is defined as follows. Consider the cup product

$$\begin{aligned} K^{g\Delta }(\mathcal {N} _{n, n+1})\times K^{\mathcal {T} _\varphi (\Delta )}(\mathcal {N} _{n, n+1})\rightarrow K^{g\Delta \cap \mathcal {T} _\varphi (\Delta )}(\mathcal {N} _{n, n+1}). \end{aligned}$$

On the other hand, there is the composition of maps

$$\begin{aligned} K^{g\Delta \cap \mathcal {T} _\varphi (\Delta )}(\mathcal {N} _{n, n+1})\xrightarrow {\textrm{nat}} K'(g\Delta \cap \mathcal {T} _\varphi (\Delta ))\xrightarrow {\chi }\mathbb {Z}. \end{aligned}$$

Here the first map is the natural map from *K*-theory to $K'$-theory (sending a complex *C* of locally free sheaves on a formal scheme *X* whose cohomology sheaves have *formal support* in a formal closed subset *Y* of *X* (i.e., which are annihilated by some power of the ideal sheaf of *Y*) to the alternating sum of the classes in $K'(Y)$ of the cohomology sheaves of *C*); the second map is given by the Euler-Poincaré characteristic (defined since $g\Delta \cap \mathcal {T} _\varphi (\Delta )$ is a proper scheme).

Combining these two maps defines the pairing

$$\begin{aligned} \langle \,, \, \rangle _{\mathcal {N} _{n, n+1}}:K^{g\Delta }(\mathcal {N} _{n, n+1})\times K^{\mathcal {T} _\varphi (\Delta )}(\mathcal {N} _{n, n+1})\rightarrow \mathbb {Z}. \end{aligned}$$

Tensoring with $\mathbb {Q}$, we obtain the pairing

6.1.2 $$\begin{aligned} \langle \,, \, \rangle _{\mathcal {N} _{n, n+1}}:K^{g\Delta }(\mathcal {N} _{n, n+1})_\mathbb {Q} \times K^{\mathcal {T} _\varphi (\Delta )}(\mathcal {N} _{n, n+1})_\mathbb {Q} \rightarrow \mathbb {Q}. \end{aligned}$$

Consider the element $[g\Delta ]\in K^{g\Delta }(\mathcal {N} _{n, n+1})$, namely the structure sheaf $\mathcal {O} _{g\Delta }$, considered as an element in $K'(g\Delta )=K^{g\Delta }(\mathcal {N} _{n, n+1})$. Similarly, consider the element $[\Delta ]\in K^{\Delta }(\mathcal {N} _{n, n+1})$ and its image $\mathbb {T} ^\Delta _\varphi ([\Delta ])\in K^{\mathcal {T} _\varphi (\Delta )}(\mathcal {N} _{n, n+1})_\mathbb {Q}$ under the map $\mathbb {T} ^\Delta _\varphi :K^{\Delta }(\mathcal {N} _{n, n+1})\rightarrow K^{\mathcal {T} _\varphi (\Delta )}(\mathcal {N} _{n, n+1})_\mathbb {Q}$. Then the RHS of (6.1.1) is defined as $\langle [g\Delta ], \mathbb {T} ^\Delta _\varphi ([\Delta ]) \rangle _{\mathcal {N} _{n, n+1}}$.

#### Remark 6.1.2

Let $\varphi$ be a monomial element. If Conjecture 5.5.1 holds true, then the quantity ${{\,\mathrm{\textrm{Int}}\,}}(g, \varphi )$ does not depend on the order in which the monomial element $\varphi$ is written as a product of atomic elements. This independence is all that matters in the formulation of the AFL conjecture. Then ${{\,\mathrm{\textrm{Int}}\,}}(g, \varphi )$ defines a linear functional $\mathcal {H} _{K^\flat \times K}\rightarrow \mathbb {Q}$.

#### Remark 6.1.3

Let $h\in \textrm{U}(W_1^\flat )(F_0)\times \textrm{U}(W_1)(F_0)$. Denote by

$$\begin{aligned}  &   h_*:K^{\Delta }(\mathcal {N} _{n, n+1})\rightarrow K^{h(\Delta )}(\mathcal {N} _{n, n+1}), \text { resp. }\,\\  &   h_*:K^{\mathbb {T} _\varphi (\Delta )}(\mathcal {N} _{n, n+1})\rightarrow K^{h(\mathbb {T} _\varphi (\Delta ))}(\mathcal {N} _{n, n+1}) \end{aligned}$$

its action on the K-group. Then $h_*\circ \mathbb {T} ^\Delta _\varphi =\mathbb {T} ^{h(\Delta )}_\varphi \circ h_*$. If *h* is a diagonal element induced by an element in $\textrm{U}(W_1^\flat )(F_0)$, then $h\Delta =\Delta$. It follows that for $h_1, h_2\in \textrm{U}(W_1^\flat )(F_0)$,

$$\begin{aligned} \begin{aligned} {{\,\mathrm{\textrm{Int}}\,}}(g,\varphi )&=\langle g\Delta , \mathbb {T} ^\Delta _\varphi (\Delta ) \rangle =\langle gh_1\Delta , \mathbb {T} ^\Delta _\varphi (h_2\Delta ) \rangle \\&=\langle gh_1\Delta , h_2\mathbb {T} ^\Delta _\varphi (\Delta ) \rangle \\  &=\langle h_2^{-1}gh_1\Delta , \mathbb {T} ^\Delta _\varphi (\Delta ) \rangle \\&={{\,\mathrm{\textrm{Int}}\,}}(h_2^{-1}gh_1,\varphi ). \end{aligned} \end{aligned}$$

Therefore the function $g\in G_{W_1}(F_0)_\textrm{rs} \mapsto {{\,\mathrm{\textrm{Int}}\,}}(g,\varphi )$ depends only on the orbit of *g*.

#### Conjecture 6.1.4

(AFL for the spherical Hecke algebra, homogeneous version.) Let $\varphi '\in \mathcal {H}_{K^{\prime \flat }}\otimes \mathcal {H}_{K'}$, and let $\varphi =\textrm{BC}(\varphi ')\in \mathcal {H}_{K^\flat }\otimes \mathcal {H}_K$. Then

$$\begin{aligned} 2 {{\,\mathrm{\textrm{Int}}\,}}(g,\varphi )\cdot \log q= -\omega (\gamma )\partial \!\operatorname {Orb}\big (\gamma , \varphi '\big ), \end{aligned}$$

whenever $\gamma \in G'(F_0)_\textrm{rs}$ is matched with $g\in G_{W_1}(F_0)_\textrm{rs}$.

In §7 we prove the conjecture when $n=1$, cf. Theorem 8.2.3.

#### Remark 6.1.5

To ensure that the LHS is well-defined, we are implicitly using the commutativity conjecture, Conjecture 5.5.1 (for the product $\mathcal {N} _{n, n+1}$, cf. Sect. 5.6) or its weakened version, cf. Remark 6.1.2. However, one may bypass this by interpreting Conjecture 6.1.4 as saying that the AFL identity holds for any representative $\widetilde{\varphi }\in \widetilde{\mathcal {H}}_K$ of $\varphi$ in the noncommutative polynomial algebra corresponding to the polynomial basis given by the atomic generators, cf. (5.5.4).

### The inhomogeneous version of the AFL

This is the special case when $\varphi =\textbf{1}_{K^\flat }\otimes f$. Moreover we choose $\varphi '=\textbf{1}_{K'^\flat }\otimes f'$ for $f'\in \mathcal {H} _{K'}$ with $\textrm{BC}_{n+1}(f')=f$. In this case it is more elegant to formulate the analytic side in terms of the symmetric space $S_{n+1}$. In fact, by Lemma 3.7.2 we have for $\gamma =(\gamma _1,\gamma _2)\in G'(F_0)_\textrm{rs}$,

$$\begin{aligned} \omega _{G'}(\gamma ) \partial \!\operatorname {Orb}\bigl (\gamma ,\textbf{1}_{K'^\flat }\otimes f'\bigr )=2\omega _{S}(r(\gamma _1^{-1}\gamma _2)) \partial \!\operatorname {Orb}(r(\gamma _1^{-1}\gamma _2),r_{*}^{\eta ^n}(f')). \end{aligned}$$

By Remark 6.1.3, it suffices to consider the regular semi-simple elements of the form (1, *g*), with $g\in \textrm{U}(W_1)(F_0)_\textrm{rs}$.

Recall from (3.7.3) and (3.7.5) the isomorphismThen the inhomogeneous version AFL is as follows. We emphasize that this conjecture is only a special case of Conjecture 6.1.4.

#### Conjecture 6.2.1

(AFL for the spherical Hecke algebra $\mathcal {H} _K$, inhomogeneous version.) Let $f\in \mathcal {H}_K$. Then

$$\begin{aligned} {{\,\mathrm{\textrm{Int}}\,}}((1,g), \textbf{1}_{K^\flat }\otimes f)\cdot \log q= -\omega (\gamma )\partial \!\operatorname {Orb}\big (\gamma , (\textrm{BC}^{\eta ^n}_{S_{n+1}})^{-1}(f)\big ), \end{aligned}$$

whenever $g\in \textrm{U}(W_1)_{\textrm{rs}}$ is matched with $\gamma \in S_{n+1}(F_0)_\textrm{rs}$.

#### Remark 6.2.2

There is also the special case of the AFL conjecture 6.1.4 when the second factor of $\varphi \in \mathcal {H}_{K^\flat }\otimes \mathcal {H}_K$ is the unit element. It is not clear to us how to simplify the analytic side in this case.

## The case $n=1$

In this section we provide evidence to Conjecture 6.1.4 by proving the case $n=1$. We also give a direct (local) proof of the FL for the full spherical Hecke algebra in this case (cf. Sect. 3.6). We use the following notation. Let $G'=\textrm{GL}_2(F)$ and $K'=\textrm{GL}_2(O_F)$. We write $\varpi ^{(m, m')}$ for the diagonal matrix in $G'$ with entries $\varpi ^{m}$ and $\varpi ^{m'}$. Also, $G=\textrm{U}(W_0)$, where we use the Hermitian form on the standard 2-dimensional vector space given by the Hermitian matrix

7.0.1 $$\begin{aligned} \begin{pmatrix} &  \sqrt{\epsilon }\\ -\sqrt{\epsilon } &  \end{pmatrix}, \quad \epsilon \in O^\times _{F_0}\setminus O^{\times , 2}_{F_0}. \end{aligned}$$

We let $K=G\cap \textrm{M}_2(O_F)$ be the natural hyperspecial maximal compact subgroup.

### Base change homomorphism

In this subsection we explicate the base change homomorphism

$$\begin{aligned} \textrm{BC}:\mathcal {H}_{K'}\rightarrow \mathcal {H} _K, \end{aligned}$$

as well as an auxiliary map and its factorization (3.7.8), which we record here for later use,7.1.1Our goal is to compute the isomorphism $\textrm{BC}^\eta _{S}$ explicitly in terms a certain basis of $\mathcal {H} _K$. We will use freely statements from Sect. 3.

Set

$$\begin{aligned} f_m'=\textbf{1}_{ M_2(O_F)_{v\circ \det =m}},\quad m\ge 0. \end{aligned}$$

The Satake isomorphism $\mathcal {H}_{K'}\simeq \mathbb {C} [X,Y]^{W}$ for $\textrm{GL}_2(F)$ is explicitly given by

7.1.2 $$\begin{aligned} \textrm{Sat}( f'_m)=q^{m}\frac{X^{m+1}-Y^{m+1}}{X-Y}. \end{aligned}$$

Here and below we use $X=x_1, Y=x_2$ for $x_1,x_2$ in Sect. 3.

Similarly, setting

$$\begin{aligned} f_m=\textbf{1}_{ \varpi ^{-m}M_2(O_F)\cap G}, \end{aligned}$$

the Satake isomorphism for the unitary group is explicitly given by

7.1.3 $$\begin{aligned} \textrm{Sat}( f_m)=q^{m}\frac{X^{(2m+1)/2}-Y^{(2m+1)/2}}{X^{1/2}-Y^{1/2}}, \end{aligned}$$

where $XY=1$. Note that, despite the fractional exponents, the last expression is a polynomial of *X* and $Y=X^{-1}$:

7.1.4 $$\begin{aligned} \textrm{Sat}( f_m)=q^{m}\sum _{i=-m}^m X^{i}. \end{aligned}$$

Set

7.1.5 $$\begin{aligned} \phi _m:=\textbf{1}_{K\varpi ^{(m,-m)} K}=f_m-f_{m-1}. \end{aligned}$$

Here, and the sequel, we set $f_m'=0$ for $m<0$, and similarly for $f_m$. Then we have a basis of $\mathcal {H} _K$ given by $\phi _m, m\ge 0$. In terms of the functions introduced in Sects. 3 and 4, we have $\phi _1=f^{[2]}$, cf. (3.5.1), hence $\phi _1=\varphi _2-(q+1)$, cf. (4.1.10).

The base change homomorphism is then the natural quotient map defined by setting $XY=1$:

7.1.6 $$\begin{aligned} \textrm{BC}:\mathbb {C} [X,Y]^{W}\rightarrow \mathbb {C} [X, X^{-1}]^{W}\simeq \mathbb {C} [X+X^{-1}]. \end{aligned}$$

Next we describe explicitly the other two maps in the diagram (7.1.1). Consider the Cartan decomposition for the symmetric space $S_2$,

$$\begin{aligned} S_2(F_0)=\coprod _{m\ge 0} {K'}\cdot \begin{pmatrix} &  \varpi ^{m}\\ \varpi ^{-m} &  \end{pmatrix}. \end{aligned}$$

The subset indexed by $m=0$ is $K'_S=K'\cdot 1_2=K'\cap S_2(F_0)$. We denote the complete set of representatives of ${K'}$-orbits on $S_2(F_0)$,

7.1.7 $$\begin{aligned} t_m:= \begin{pmatrix} &  \varpi ^m\\ \varpi ^{-m} &  \end{pmatrix},\quad m\ge 0. \end{aligned}$$

Therefore, we have a basis of $\mathcal {H} _{K'_S}$ given by

7.1.8 $$\begin{aligned} \varphi '_m=\textbf{1}_{{K'}\cdot t_m},\quad m\ge 0. \end{aligned}$$

Recall the map $r^\eta _*$ from (3.7.7). Note that a basis of $\mathcal {H}_{K'}$ is given by $\textbf{1}_{\varpi ^j {K'}\varpi ^{(m,0)}{K'}}, j\in \mathbb {Z},m\ge 0$.

#### Lemma 7.1.1

(i) The map $r^\eta _*:\mathcal {H}_{K'}\rightarrow \mathcal {H} _{K'_S}$ sends $\textbf{1}_{\varpi ^j {K'}\varpi ^{(m,0)}{K'}}$ to $(-1)^m\sum _{i=0}^m e_{m-i} \varphi '_{i}$, where
  $$\begin{aligned} e_i=\left\{ \begin{array}{ll} 1, & i=0,\\ q^{i}(1+q^{-1}), &  i>0. \end{array}\right. \end{aligned}$$
(ii) The map $r^\eta _*$ sends $f'_m$ to $(-1)^m\sum _{i=0}^m (\sum _{j=0}^{m-i} q^j ) \varphi '_{i}.$
(iii) We have $\textrm{BC}^\eta _S({\tilde{\varphi }}'_{m})=\phi _{m}$, where
  7.1.9 $$\begin{aligned} {\tilde{\varphi }}'_{m}:= (-1)^m( \varphi '_m+2\varphi '_{m-1}+\cdots +2 \varphi '_0)\in \mathcal {H} _{K'_S}. \end{aligned}$$

#### Proof

We first show part (ii). We need to compute the integral

$$\begin{aligned} r^\eta _*(f'_m)(g\overline{g}^{-1})=\int _{\textrm{GL}_2(F_0)} f'_m(gh)\widetilde{\eta }(gh)\, dh. \end{aligned}$$

Since the determinant of any element in the support of $f'_m$ has valuation *m*, the integral is equal to

$$\begin{aligned} r^\eta _*(f'_m)(g\overline{g}^{-1})=(-1)^m\int _{\textrm{GL}_2(F_0)} f'_m(gh)\, dh. \end{aligned}$$

It suffices to determine its value at elements of the form $t_\ell$, cf. (7.1.7). By Iwasawa decomposition, every element in $\textrm{GL}_2(F)$ lies in in some $${K'}\begin{pmatrix} 1&  u\\ &  1 \end{pmatrix} \begin{pmatrix} \varpi ^{m-i}& \\ &  \varpi ^i \end{pmatrix} $$. All elements in this $K'$-coset are mapped into a single ${K'}$-orbit in $S_2(F_0)$ with representative

$$\begin{aligned} \begin{pmatrix} 1&  u\\ &  1 \end{pmatrix} \begin{pmatrix} 1&  \overline{u}\\ &  1 \end{pmatrix}^{-1} = \begin{pmatrix} 1&  u-\overline{u}\\ &  1 \end{pmatrix}. \end{aligned}$$

This last element is ${K'}$-equivalent (in $S_2(F_0)$) to $t_{\max \{-v(u-\overline{u}),0\}}$ (recall that we are assuming that the residue characteristic is odd). Therefore it suffices to compute the integral for $$g=\begin{pmatrix} 1&  u\\ &  1 \end{pmatrix}$$, where $u\in F^{-}$ lies in the purely imaginary part and has valuation $v(u)\le 0$.

We use the Iwasawa decomposition $\textrm{GL}_2(F_0)=ANK_0$, where *A* denotes the diagonal torus, *N* the subgroup of upper triangular unipotent matrices, and $K_0=\textrm{GL}_2(O_{F_0})$. Accordingly we write an element in $\textrm{GL}_2(F_0)$ as $$h=\begin{pmatrix} x&  \\ &  y \end{pmatrix}\begin{pmatrix} 1&  z \\ &  1 \end{pmatrix} k$$. The Haar measure on $\textrm{GL}_2(F_0)$ is then given by $d^\times x \, d^\times y\,dz$, where the multiplicative (resp. additive) Haar measure on $F_0^\times$ (resp. $F_0$) is normalized such that ${{\,\textrm{vol}\,}}(O_{F_0}^\times )=1$ (resp. ${{\,\textrm{vol}\,}}(O_{F_0})=1$). Then the condition $gh\in M_2(O_F), v(\det (gh))=m$ is equivalent to

$$\begin{aligned} x,y\in O_{F_0}, \quad v(xy)=m,\quad xz\in O_{F_0},\quad y u\in O_{F} \end{aligned}$$

(note that $x,y,z\in F_0$ and $u\in F^{-}$). It follows that

$$\begin{aligned} \int _{\textrm{GL}_2(F_0)} f'_m(\begin{pmatrix} 1&  u\\ &  1 \end{pmatrix} h)\, dh= \int _{-v(u)\le v(y)\le m} \int _{v(x)=m-v(y)} \int _{z\in \frac{1}{x}O_{F_0}} dz \,d^\times x \, d^\times y. \end{aligned}$$

This triple integral is equal to

$$\begin{aligned} \int _{\textrm{GL}_2(F_0)} f'_m(\begin{pmatrix} 1&  u\\ &  1 \end{pmatrix} h)\, dh=\sum _{0\le i\le m+v(u)} q^i. \end{aligned}$$

We thus obtain the value of $r^\eta _*(f_m')$ at the element $t_\ell$ in (7.1.7),

$$\begin{aligned} r^\eta _*(f_m')(t_{\ell })=(-1)^m\left\{ \begin{array}{ll}\sum _{0\le i\le m-\ell } q^i,& 0\le \ell \le m, \\ 0, & \ell >m. \end{array}\right. \end{aligned}$$

A comparison with (7.1.8) yields

$$\begin{aligned} r^\eta _*(f_m')=(-1)^m \sum _{i=0}^m \left( \sum _{j=0}^{m-i} q^j \right) \varphi '_{i}. \end{aligned}$$

This shows part (ii). To show part (i), by $\textbf{1}_{K' \varpi ^{(m,0)}K'} =f'_m-f'_{m-2}$, we deduce from part (ii) that

$$\begin{aligned} r^\eta _*(\textbf{1}_{K' \varpi ^{(m,0)}K'})=r^\eta _*( f'_m-f'_{m-2})=\sum _{i=0}^m e_{m-i} \varphi '_{i}. \end{aligned}$$

It remains to show part (iii). From the explicit formulas of Satake transforms (7.1.2) and (7.1.3), we see that the map $$\textrm{BC}$$ in (7.1.6) sends $$f_m'+q f'_{m-1}$$ to $$ f_m$$. It follows that

7.1.10 $$\begin{aligned} \textrm{BC}(f_m'+(q-1) f'_{m-1}-f'_{m-2} )=\phi _m,\quad m\ge 0. \end{aligned}$$

From part (ii) we have

$$\begin{aligned} r^\eta _*(f_m'+(q-1) f'_{m-1}-f'_{m-2})={\tilde{\varphi }}'_{m}. \end{aligned}$$

By the commutative diagram (7.1.1), we see that $\textrm{BC}_S^\eta$ sends ${\tilde{\varphi }}'_{m}$ to $\phi _m$, as desired.

$$\square $$

#### Remark 7.1.2

The explicit description above gives a direct proof of (3.7.3) in the case $n=2$.

### Orbital integrals on the unitary group

We now consider the orbital integrals of elements in $\mathcal {H} _{K}$. Recall from (7.1.5) that $\phi _{m}$ denotes $\textbf{1} _{K \varpi ^{(m, -m)}K}$, where, we recall, *K* denotes the hyperspecial subgroup of $U(W_0)$. Now we make a change of Hermitian form for $W_0$ from (7.0.1) to the identity matrix. This is for the convenience of orbit comparison in (3.3.2). We define the invariants map on the orbits of $G(F_0)$ by

7.2.1 $$\begin{aligned} g= \begin{pmatrix} a &  b\\ c &  d \end{pmatrix}\mapsto (a,d, bc), \end{aligned}$$

(note that (*a*, *d*, *bc*) are not independent).

#### Proposition 7.2.1

Let $m\ge 1$. Then for $$g= \begin{pmatrix} a &  b\\ c &  d \end{pmatrix}\in G(F_0)$$ regular semisimple, we have

7.2.2 $$\begin{aligned} {{\,\textrm{Orb}\,}}(g, \phi _m)= \left\{ \begin{array}{ll} 1, &  v(1-a\overline{a})=-2m\\ 0, &  v(1-a\overline{a})\ne -2m. \end{array}\right. \end{aligned}$$

#### Proof

We have, since we use the Hermitian form on $W_0$ given by the identity matrix,

7.2.3 $$\begin{aligned} a{\bar{a}}=d{\bar{d}}=1-b{\bar{b}}, \quad a{\bar{c}}=b{\bar{d}}\ne 0. \end{aligned}$$

The invariants are *a*, *d* and $bc=(1-a\overline{a})d/\overline{a}$. Since the group $H=\textrm{U}_1(F_0)$ is compact and lies in *K*, the orbital integral is either 1 or 0, depending on whether *g* lies in the support of $\phi _m$ or not. We note that the support of $\phi _m$ is the set of matrices $g\in G(F_0)$ such that $\varpi ^m g\in M_2(O_F)$ and such that there exists at least one entry of *g* with valuation exactly $-m$. With the help of the above identities, it is easy to see that the support condition amounts to *a*, *b*, *c*, *d* all having valuation equal to $-m$. $\square$

### Orbital integrals on the symmetric space $S_2$

Recall that the inhomogeneous orbital integral is defined by (3.2.3). We also recall from [31, Sect. 15.1] the structure of regular semisimple sets on the symmetric space $S(F_0) = S_2(F_0)$. We write an element as

7.3.1 $$\begin{aligned} \gamma = \begin{pmatrix} a &  b \\ c &  d \end{pmatrix}\in S(F_0). \end{aligned}$$

Then $\gamma$ is regular semi-simple if and only if $bc\ne 0$, in which case we may write $\gamma$ as

7.3.2 $$\begin{aligned} \begin{aligned} \gamma =\gamma (a,b)&:=\begin{pmatrix} a &  b \\ (1-\textrm{N} a)/\overline{b} &  -\overline{a}b/\overline{b} \end{pmatrix}\\&=\begin{pmatrix} 1&  \\ &  -b/\overline{b} \end{pmatrix} \begin{pmatrix} a &  b \\ - (1-\textrm{N} a)/ b &  \overline{a} \end{pmatrix} \in S(F_0)_\textrm{rs}, \quad a \in F \smallsetminus F^1,\ b \in F^\times . \end{aligned} \end{aligned}$$

Similarly to the unitary group case, we define the invariant map on the orbits of $S(F_0)$,

7.3.3 $$\begin{aligned} \gamma = \begin{pmatrix} a &  b\\ c &  d \end{pmatrix}\mapsto (a,d, bc), \end{aligned}$$

(again, (*a*, *d*, *bc*) are not independent, cf. (7.2.1)). Then an orbit of $\gamma \in S(F_0)_\textrm{rs}$ matches an orbit of $g\in G(F_0)_\textrm{rs}$ if and only if they have the same invariants. In particular, an element $\gamma \in S(F_0)_\textrm{rs}$ matches an element in the quasi-split (resp. non-quasi-split) unitary group if and only if $v(1- a{\bar{a}})$ is even (resp. $v(1-a{\bar{a}})$ is odd).

We now consider the orbital integral of elements in $\mathcal {H} _{K'_S}$. Since $F/F_0$ is unramified, we may assume that, up to conjugation by $H=\textrm{GL}_1(F_0)$, for a regular semisimple $\gamma$ as in (7.3.1) that the entry *c* is a unit.

#### Proposition 7.3.1

Let $$\gamma = \begin{pmatrix} a &  b\\ c &  d \end{pmatrix}\in S_2(F_0)$$ be regular semisimple with $v(c)=0$. When $m\ge 1$, we have7.3.4When $m=0$, we have

7.3.5 $$\begin{aligned} {{\,\textrm{Orb}\,}}(\gamma , \varphi '_0,s)= \left\{ \begin{array}{ll} \sum _{i=0}^{v(1-a\overline{a})} (-1)^i q^{-is}, &  v(1-a\overline{a})\ge 0\\ 0, &  v(1-a\overline{a})<0 . \end{array}\right. \end{aligned}$$

#### Proof

Let $$\gamma = \begin{pmatrix} a &  b\\ c &  d \end{pmatrix}\in S_2(F_0)$$ be regular semisimple. Then we have

7.3.6 $$\begin{aligned} 1-a{\bar{a}}=1-d{\bar{d}}= c{\bar{b}}=b{\bar{c}}\ne 0. \end{aligned}$$

Assume $m>0$. We first consider those orbits $\gamma$ such that $a\in O_F$. Then $d\in O_F$ and $b{\bar{c}}\in O_F$. Then the condition for $$h^{-1}\gamma h=\begin{pmatrix} a &  h^{-1}b\\ ch &  d \end{pmatrix}$$ to be in ${{\,\textrm{supp}\,}}(\varphi '_m)$ is that either $h^{-1}b$ or *ch* lies in $\varpi ^{-m}O_F^\times$. It follows that $v(h)\in \{-m, v(bc)+m\}$ and

7.3.7 $$\begin{aligned} {{\,\textrm{Orb}\,}}(\gamma , \varphi '_m,s)= (-1)^m q^{ms}(1+\eta (b{\bar{c}})q^{-(v(b{\bar{c}})+2m)s}). \end{aligned}$$

Next we consider the orbits with $a\notin O_F$. Then $a{\bar{a}}$, $d{\bar{d}}$ and $c{\bar{b}}=b{\bar{c}}$ all have equal valuations. If $v(a)=-m$, then $v(b)=2v(a)=-2\,m$ (note that we have assumed $v(c)=0$). The condition for $h\cdot \gamma \in {K'}\cdot t_m$ is

$$\begin{aligned} v(h^{-1}b)\ge -m,\quad v(ch)=v(h)\ge -m, \end{aligned}$$

or equivalently

$$\begin{aligned} -m\le v(h)\le m+v(b)=-m. \end{aligned}$$

Therefore the orbital integral is equal to

7.3.8 $$\begin{aligned} {{\,\textrm{Orb}\,}}(\gamma , \varphi '_m,s)= (-1)^m q^{ms}. \end{aligned}$$

If $-m<v(a)<0$, then $v(b)=2v(a)\ne -2\,m$. A similar argument shows that $v(h)\in \{-m, m+v(b)\}$ and

7.3.9 $$\begin{aligned} {{\,\textrm{Orb}\,}}(\gamma , \varphi '_m,s)= (-1)^m q^{ms} (1+\eta (b{\bar{c}})q^{-(v(b{\bar{c}})+2m)s}). \end{aligned}$$

We have thus proved (7.3.4).

Assume $m=0$. If $v(a)\ge 0$, then

7.3.10 $$\begin{aligned} {{\,\textrm{Orb}\,}}(\gamma , \varphi '_0,s)= \sum _{i=0}^{v(bc)} (-1)^i q^{-is}. \end{aligned}$$

The case $v(a)<0$ is similar to the $m>0$ case.

Finally note that $v(b)=v(bc)=v(1-a\overline{a})$. The proof is complete. $\square$

#### Proposition 7.3.2

(i) The functions $\phi _{m}\in \mathcal {H} _K$ and ${\tilde{\varphi }}'_{m}\in \mathcal {H} _{K'_S}$ are transfers of each other.
(ii) Let $$\gamma = \begin{pmatrix} a &  b\\ c &  d \end{pmatrix}\in S_2(F_0)_\textrm{rs} $$ with $v(c)=0$. Assume $v(1-a\overline{a})$ odd (so that $\gamma$ matches an element in $\textrm{U}(W_1)(F_0)_\textrm{rs}$). When $m\ge 1$, we have
  $$\begin{aligned} \partial \!\operatorname {Orb}(\gamma ,{\tilde{\varphi }}'_{m})= \log q \left\{ \begin{array}{ll} 1, &  v(1-a\overline{a})> 0\\ 0, &  v(1-a\overline{a})<0. \end{array}\right. \end{aligned}$$
  When $m=0$, we have
  $$\begin{aligned} \partial \!\operatorname {Orb}(\gamma ,{\tilde{\varphi }}'_0)= \log q \left\{ \begin{array}{ll} \frac{v(1-a\overline{a})+1}{2}, &  v(1-a\overline{a})\ge 0\\ 0, &  v(1-a\overline{a})<0. \end{array}\right. \end{aligned}$$

#### Proof

When $m>0$, we have the value at $s=0$,

7.3.11 $$\begin{aligned} {{\,\textrm{Orb}\,}}(\gamma , \varphi '_m)=(-1)^m \left\{ \begin{array}{ll} 1+\eta (1-a\overline{a}), &  v(1-a\overline{a})> -2m,\\ 1, &  v(1-a\overline{a})=-2m,\\ 0, &  v(1-a\overline{a})<-2m, \end{array}\right. \end{aligned}$$

and, when $v(1-a\overline{a})$ is odd, we have the first derivative at $s=0$,

$$\begin{aligned} \partial \!\operatorname {Orb}(\gamma ,\varphi '_{m})= (-1)^m\log q \left\{ \begin{array}{ll} v(1-a\overline{a})+2m, &  v(1-a\overline{a})> -2m\\ 0, &  v(1-a\overline{a})<-2m. \end{array}\right. \end{aligned}$$

When $m=0$, we have the value at $s=0$,

7.3.12 $$\begin{aligned} {{\,\textrm{Orb}\,}}(\gamma , \varphi '_0)=(-1)^m \left\{ \begin{array}{ll} 1, &  v(1-a\overline{a})\ge 0 \\ 0, &  v(1-a\overline{a})<0 , \end{array}\right. \end{aligned}$$

and, when $v(1-a\overline{a})$ is odd, we have the first derivative at $s=0$,

$$\begin{aligned} \partial \!\operatorname {Orb}(\gamma ,\varphi '_0)= (-1)^m\log q \left\{ \begin{array}{ll} \frac{v(1-a\overline{a})+1}{2}, &  v(1-a\overline{a})\ge 0\\ 0, &  v(1-a\overline{a})<0. \end{array}\right. \end{aligned}$$

Hence, when $v(1-a\overline{a})=-2m$, we have ${{\,\textrm{Orb}\,}}(\gamma , \widetilde{\varphi }'_m)={{\,\textrm{Orb}\,}}(\gamma , \varphi '_m)=1$ (cf. the definition of $\widetilde{\varphi }'_m$ in (7.1.9)). When $v(1-a\overline{a})=-2(m-i)>-2m$ is even, we have from (7.3.11) and (7.3.12)

$$\begin{aligned} {{\,\textrm{Orb}\,}}(\gamma , {\tilde{\varphi }}'_m)=2-4+4+\cdots +(-1)^{i-1}4+(-1)^i2=0, \end{aligned}$$

It follows from a comparison with Proposition 7.2.1 that $\phi _{m}\in \mathcal {H} (U(V))$ and ${\tilde{\varphi }}'_{m}\in \mathcal {H} (S_2)$ are transfers of each other. This proves part (i).

It remains to compute the first derivative $\partial \!\operatorname {Orb}(\gamma ,\widetilde{\varphi }'_{m})$ when $v(1-a\overline{a})>-2m$ is odd. It suffices to consider the case when $v(1-a\overline{a})=v(bc)$ is positive. Set $v(1-a\overline{a})=-2(m-i)+1$. Then $\partial \!\operatorname {Orb}(\gamma ,{\tilde{\varphi }}'_{m})$ equals $\log q$ times

$$\begin{aligned}&(v(bc)+2m)-2(v(bc)+2(m-1))+\cdots +(-1)^{m-1}2(v(bc)+2)\\&\qquad +(-1)^m(v(bc)+1)\\&\quad =(2m-2(m-1))-\cdots +(-1)^{i-1}(2(m-i+1)-2(m-i))\\&\qquad +\cdots +(-1)^{m-1}(2-1)\\&\quad =2-2+2-\cdots +(-1)^{m-2}2+(-1)^{m-1}\\&\quad =1. \end{aligned}$$

The proof is complete. $\square$

#### Remark 7.3.3

Part (i) gives a direct proof in the case $n=1$ of the FL for the full spherical Hecke algebra, cf. §3.6 and §3.7.

### Intersection numbers

In this subsection, we often write $\mathcal {N} ^{[0]}$, or simply $\mathcal {N}$, for $\mathcal {N} _2^{[0]}$. Note that $\mathcal {N} \simeq {{\,\textrm{Spf}\,}}(W[[t]])$ has only one point. Recall the Hecke operator $\mathbb {T} ^A_{\varphi _2}:K^A(\mathcal {N})\rightarrow K^{\mathcal {T} _{\le 2}(A)}(\mathcal {N})$. It is defined as $\mathbb {T} ^A_{\varphi _2}=\mathbb {T} ^A_{\le 2}=\mathbb {T} ^{\mathcal {N} ^{[0,2]}(A),-}\circ \mathbb {T} ^{A, +}$, where

7.4.1 $$\begin{aligned} \begin{aligned} \mathbb {T} ^{A,+}&=(\mathcal {N} ^{[0,2]})_*:K^A(\mathcal {N} _2^{[0]})\rightarrow K^{\mathcal {N} ^{[0,2]}(A)}(\mathcal {N} _2^{[2]}), \\ \mathbb {T} ^{B,-}&=(\mathcal {N} ^{[2, 0]})_*:K^B(\mathcal {N} _2^{[2]})\rightarrow K^{\mathcal {N} ^{[2,0]}(B)}(\mathcal {N} _2^{[0]}), \end{aligned} \end{aligned}$$

cf. (5.5.3). We may identify $\mathcal {N} _2^{[2]}$ with $\mathcal {N}$ (cf. Remark 5.5.6), and then both Hecke operators in (7.4.1) are induced by the same geometric correspondence $\mathcal {T} _{\Gamma _0}\rightarrow \mathcal {N} \times \mathcal {N}$ and its transpose, i.e., we can write

7.4.2 $$\begin{aligned} \mathcal {T} _{\le 2}=\mathcal {T} _{\Gamma _0}\circ {^t\mathcal {T} _{\Gamma _0}}. \end{aligned}$$

The notation $\mathcal {T} _{\Gamma _0}$ is a reminder of the fact that $\mathcal {T} _{\Gamma _0}$ is the analogue of the $\Gamma _0(p)$-covering of the modular curve in the present context, cf. Remark 5.5.6.

Since the projection morphisms from the composition $\mathcal {T} _{\le 2}$ of the geometric correspondences $\mathcal {N} ^{[0,2]}$ and $\mathcal {N} ^{[2, 0]}$ to $\mathcal {N}$ are finite and flat, we have $\mathbb {T} _{\le 2}=(\mathcal {T} _{\le 2})_*$, cf. Lemma 9.2.1 (we are leaving out the support *A* from the notation). A similar reasoning shows that $\mathbb {T} _{\varphi _2^m}=((\mathcal {T} _{\le 2})^m)_*$, where $(\mathcal {T} _{\le 2})^m=\mathcal {T} _{\le 2}\circ \cdots \circ \mathcal {T} _{\le 2}$ is the *m*-fold iterated composition of $\mathcal {T} _{\le 2}$ with itself (which again maps by finite flat morphisms to $\mathcal {N} ^{[0]}$).

At this point we use the local intersection calculus developed in [23, Sect. 5] (the role of the regular local formal scheme *M* in loc. cit. is played here by $\mathcal {N}$).

We consider the natural morphism $(\mathcal {T} _{\le 2})^m\rightarrow \mathcal {N} \times \mathcal {N}$. Since $(\mathcal {T} _{\le 2})^m$ is finite and flat over $\mathcal {N}$, this morphism is finite with image of codimension one. We associate to it an element of the group $Z^1(\mathcal {N} \times \mathcal {N})$ of cycles of codimension one (namely $\sum _{Z\in (\mathcal {N} \times \mathcal {N})^{(1)}}\ell _{\mathcal {O} _{\eta _Z}}(\mathcal {O} _{(\mathcal {T} _{\le 2})^m})[Z]\in Z^1(\mathcal {N} \times \mathcal {N})$, cf. [23, Def. 5.1]). We use the same notation $(\mathcal {T} _{\le 2})^m$ for this one-cycle. Since $\mathcal {N} \times \mathcal {N}$ is regular, we may regard $(\mathcal {T} _{\le 2})^m$ as an effective Cartier divisor on $\mathcal {N} \times \mathcal {N}$. By the flatness of $(\mathcal {T} _{\le 2})^m$ over $\mathcal {N}$, it is in fact a relative Cartier divisor, i.e., it is at every point defined by one equation in $W[[t, t']]$ which is neither a unit nor divisible by *p*.

Both projection maps to $\mathcal {N}$ are finite, hence $(\mathcal {T} _{\le 2})^m$ is an element of the ring of correspondences $\textrm{Corr}(\mathcal {N})$, and from the definition of the ring structure on $\textrm{Corr}(\mathcal {N})$, it follows that $(\mathcal {T} _{\le 2})^m$ is the *m*-th power of the correspondence $\mathcal {T} _{\le 2}$, cf. [23, Def. 5.6]. We obtain a homomorphism of $\mathbb {Q}$-algebras,

$$\begin{aligned} \mathcal {H} _K\rightarrow \textrm{Corr}(\mathcal {N} \times \mathcal {N})_\mathbb {Q},\quad \sum _m a_m\varphi _2^m\mapsto \sum _m a_m(\mathcal {T} _{\le 2})^m. \end{aligned}$$

We denote the image of $\varphi$ under this map by $\mathcal {T} _{\varphi }$. Note that the unit element $\varphi =\textbf{1}$ is mapped to $\mathcal {T} _\textbf{1}=\Delta _{\mathcal {N} \times \mathcal {N}}$ (the diagonal in $\mathcal {N} \times \mathcal {N}$), which we denote by $\mathcal {T} _0$. To simplify the notation, we write $\mathcal {T} _1$ for $\mathcal {T} _{\varphi _2}$ (not to be confused with the divisor associated to the unit element). Also, for $\phi _{m}=\textbf{1}_{K \varpi ^{(m,-m)} K }$, we use the notation $\mathcal {T} ^\circ _{m}$ for $\mathcal {T} _{\phi _m}$. Hence $\mathcal {T} ^\circ _{0}=\mathcal {T} _{0}$ is the unit element in $\textrm{Corr}(\mathcal {N} \times \mathcal {N})_\mathbb {Q}$.

#### Lemma 7.4.1

There is the relation of Cartier divisors on $\mathcal {N} \times \mathcal {N}$,

$$\begin{aligned} \mathcal {T} ^\circ _{1}=\mathcal {T} _1-(q+1)\mathcal {T} _0, \end{aligned}$$

and the recursive relation

$$\begin{aligned} \mathcal {T} _{\varphi _2*\phi _m}=\mathcal {T} _{1}\circ \mathcal {T} ^\circ _{m}=\mathcal {T} ^\circ _{m+1} +2q \mathcal {T} ^\circ _{m}+q^2\mathcal {T} ^\circ _{m-1}, \quad m\ge 1. \end{aligned}$$

#### Proof

This follows from the relations in the Hecke algebra:

$$\begin{aligned} \phi _1=\varphi _2-(q+1), \quad \varphi _2\phi _m=\phi _{m+1}+2q\phi _m+q^2\phi _{m-1}, m\ge 1. \end{aligned}$$

The first identity is mentioned below (7.1.5). The second identity follows from this, after expressing $\phi _m$ in terms of $f_m$ as in (7.1.5) and applying the Satake isomorphism, cf. (7.1.4). $\square$

Recall the action of $\textrm{Corr}(\mathcal {N})$ on $Z^1(\mathcal {N})$, cf. [23, Def. 5.7]

7.4.3 $$\begin{aligned} \mathcal {Z} \mapsto \mathcal {T} *\mathcal {Z}. \end{aligned}$$

Hence we obtain an action of $\mathcal {H} _K$ on $Z^1(\mathcal {N})_\mathbb {Q}$.

For a non-zero $u\in \mathbb {V} _2$ we let $\mathcal {Z} (u)$ be the associated special divisor and $\mathcal {Z} (u)^\circ =\mathcal {Z} (u)-\mathcal {Z} (\varpi ^{-1}u)$ the difference divisor, cf. [34]. Both are effective Cartier divisors on $\mathcal {N}$. Recall the special vector $u_0\in \mathbb {V} _2$, cf. Sect. 6.1. It is of valuation zero (i.e., its length is a unit), and $\mathcal {Z} (u_0)^\circ =\mathcal {Z} (u_0)$.

#### Proposition 7.4.2

Let $m\ge 0$. Then there is an equality of Cartier divisors on $\mathcal {N}$,

$$\begin{aligned} \mathcal {T} ^\circ _{m}*\mathcal {Z} (u_0)= \mathcal {Z} (\varpi ^m u_0)^\circ . \end{aligned}$$

(For $m\ge 1$, both sides have degree $q^{2\,m-1}(q+1)$ over ${{\,\textrm{Spf}\,}}O_{\breve{ F}}$.)

#### Proof

By Lemma 7.4.1, this identity is equivalent to the conjunction of the following identities.

1. $$\mathcal {T} _1*\mathcal {Z}(u_0)=\mathcal {Z}(\varpi u_0)^\circ +(q+1)\mathcal {Z}(u_0).$$
2. $$\mathcal {T} _{1}*\mathcal {Z} (\varpi ^m u_0)^\circ =\mathcal {Z} (\varpi ^{m+1} u_0)^\circ +2q \mathcal {Z} (\varpi ^{m} u_0)^\circ + q^2\mathcal {Z} (\varpi ^{m-1} u_0)^\circ , \quad m\ge 1.$$

We first recall the theory of quasi-canonical divisors for $\mathcal {N}$, cf. [17]. Recall the framing object $\mathbb {X} =\mathbb {E} \times {\bar{\mathbb {E}}}$ (note that in loc. cit. the order of the factors is reversed, which results in slightly different formulas below). Then $u_0=\textrm{incl}_2\in \mathbb {V} _2={{\,\textrm{Hom}\,}}_{O_F}({\bar{\mathbb {E}}}, \mathbb {X})$. Let $u_1=\textrm{incl}_1\circ \Pi$, where $\Pi$ is a fixed uniformizer of the quaternion algebra $D={{\,\textrm{End}\,}}(\mathbb {E})^o$, comp. Example 5.5.6. Then  with ${\textrm{val}}(u_1)=1$.

The quasi-canonical divisor of level *s* is given as follows. Let $\mathcal {F} _s/O_{{\breve{F}}, s}$ be a quasi-canonical lift of level *s* of $\mathbb {E}$ in the sense of Gross. Here for any *n*, $O_{{\breve{F}}, n}$ denotes the ring of integers in the abelian extension of ${\breve{F}}$ corresponding to the subgroup $(O_{F_0}+\varpi ^n O_F)^\times$ of the group of units. Let $X^{(s)}=O_F\otimes _{O_{F_0}}\mathcal {F} _s$ (Serre construction). Then in [17, Sect. 6], $X^{(s)}$ is completed to an object $(X^{(s)}, \iota _{X^{(s)}}, \lambda _{X^{(s)}}, \rho _{X^{(s)}})$ of $\mathcal {N} (O_{F, s})$, and it is proved that the corresponding morphism ${{\,\textrm{Spf}\,}}O_{F, s}\rightarrow \mathcal {N}$ is a closed embedding defining a Cartier divisor $\mathcal {Z} _s$, the *quasi-canonical divisor* of level *s*. Then $\mathcal {Z} (u_0)=\mathcal {Z} _0$ and $\mathcal {Z} (u_1)=\mathcal {Z} _1$ and $\mathcal {Z} (\varpi u_0)=\mathcal {Z} _0+\mathcal {Z} _2$, cf. [17, Prop. 8.1]. The last identity can also be written as $\mathcal {Z} (\varpi u_0)^o=\mathcal {Z} _2$. More generally, $\mathcal {Z} (\varpi ^m u_0)^o=\mathcal {Z} _{2\,m}$ and $\mathcal {Z} (\varpi ^m u_1)^o=\mathcal {Z} _{2\,m+1}$, cf. loc. cit.

Let us now prove (1). Note that $\mathcal {T}_{\Gamma _0}\cap \pi _1^{-1}(\mathcal {Z}(u_0))\subseteq \mathcal {N}\times \mathcal {N}$ is the locus of isogenies $X^{(0)}\rightarrow Y$ of degree $q^2$. Recall the isogeny of quasi-canonical lifts $\psi _1: \mathcal {F} _0\rightarrow \mathcal {F} _1$, cf. [17, Lem. 6.2] (it depends on the choice of $\Pi$). By the functoriality of the Serre construction, it defines the isogeny $X^{(0)}\rightarrow X^{(1)}$ over $\mathcal {Z} (u_1)$. Hence we obtain a closed embedding $\iota :\mathcal {Z} (u_1)\subset \mathcal {T}_{\Gamma _0}\cap \pi _1^{-1}(\mathcal {Z}(u_0))$. Since $\pi _2\circ \iota$ is the natural embedding of $\mathcal {Z} (u_1)$ in $\mathcal {N}$, we obtain an inequality of Cartier divisors, $\mathcal {Z}(u_1)\le [\mathcal {T}_{\Gamma _0}]*\mathcal {Z}(u_0)$. Since both these Cartier divisors map with degree $q+1$ to ${{\,\textrm{Spf}\,}}O_{\breve{F}}$, they are equal, i.e.,

7.4.4 $$\begin{aligned} \mathcal {T}_{\Gamma _0}*\mathcal {Z}(u_0)=\mathcal {Z}(u_1). \end{aligned}$$

Similarly, $\mathcal {T}_{\Gamma _0}\cap \pi _1^{-1}(\mathcal {Z}(u_1))$ parametrizes isogenies $X^{(1)}\rightarrow Y$. The isogeny of quasi-canonical lifts $\psi _{1, 2}: \mathcal {F} _1\rightarrow \mathcal {F} _2$ (defined in an analogous way to $\psi _{s}: \mathcal {F} _0\rightarrow \mathcal {F} _s$ for any *s* in [17, Sect. 6]), defines the isogeny $X^{(1)}_{{{\,\textrm{Spf}\,}}O_{{\breve{F}},2}}\rightarrow X^{(2)}$. This defines a closed embedding $\mathcal {Z} _2=\mathcal {Z}(\varpi u_0)^\circ \subset \mathcal {T} _{\Gamma _0}*\mathcal {Z}(u_1)$.

On the other hand, consider the natural isogeny $X^{(0)}_{{{\,\textrm{Spf}\,}}O_{{\breve{F}},1}}\rightarrow X^{(1)}$. It induces a unique isogeny $X^{(1)}\rightarrow X^{(0)}_{{{\,\textrm{Spf}\,}}O_{{\breve{F}},1}}$ such that the composition is equal to $\varpi :X^{(1)}\rightarrow X^{(1)}$. We obtain a closed embedding of ${{\,\textrm{Spf}\,}}O_{\breve{F},1}$ into $\mathcal {T}_{\Gamma _0}\cap \pi _1^{-1}(\mathcal {Z}(u_1))$, which is mapped under $\pi _2$ onto $\mathcal {Z}(u_0)$, with degree $[O_{{\breve{F}},1}: O_{{\breve{F}}}]=q+1$. Altogether we obtain an inequality of Cartier divisors, $\mathcal {Z}(\varpi u_0)^\circ +(q+1)\mathcal {Z}(u_0)\le \mathcal {T} _{\Gamma _0}*\mathcal {Z}(u_1)$. Since both divisors have degree $(q+1)^2$ over ${{\,\textrm{Spf}\,}}O_{\breve{F}}$, we obtain the equality of divisors

7.4.5 $$\begin{aligned} \mathcal {T} _{\Gamma _0}*\mathcal {Z}(u_1)=\mathcal {Z}(\varpi u_0)^\circ +(q+1)\mathcal {Z}(u_0). \end{aligned}$$

Taking both identities (7.4.4) and (7.4.5) together, we obtain

$$\begin{aligned} \mathcal {T}_1*\mathcal {Z}(u_0)=\mathcal {T} _{\Gamma _0}*\mathcal {Z}(u_1)=\mathcal {Z}(\varpi u_0)^\circ +(q+1)\mathcal {Z}(u_0), \end{aligned}$$

which proves (1).

To prove (2), we see by the same argument that

$$\begin{aligned} \mathcal {T} _{\Gamma _0}*\mathcal {Z}(\varpi ^m u_0)^\circ =\mathcal {Z}(\varpi ^mu_1)^\circ +q\mathcal {Z}(\varpi ^{m-1}u_1)^\circ \end{aligned}$$

and

$$\begin{aligned} \mathcal {T} _{\Gamma _0}*\mathcal {Z}(\varpi ^m u_1)^\circ =\mathcal {Z}(\varpi ^{m+1}u_0)^\circ +q\mathcal {Z}(\varpi ^{m}u_0)^\circ . \end{aligned}$$

Thus

$$\begin{aligned} \mathcal {T} _1*\mathcal {Z}(\varpi ^m u_0)^\circ =\mathcal {Z} (\varpi ^{m+1} u_0)^\circ +2q \mathcal {Z} (\varpi ^{m} u_0)^\circ + q^2\mathcal {Z} (\varpi ^{m-1} u_0)^\circ , \end{aligned}$$

as desired. $$\square $$

Let $\mathcal {Z}$ and $\mathcal {Z} '$ be formal schemes finite over a closed formal subscheme of codimension one in $\mathcal {N}$, with classes $[\mathcal {Z} ]\in K^{\mathcal {Z}}(\mathcal {N})$, resp. $[\mathcal {Z} ']\in K^{\mathcal {Z} '}(\mathcal {N})$. Let $\mathcal {T} \rightarrow \mathcal {N} \times \mathcal {N}$ be a correspondence. Assume that $\mathcal {Z}\cap \mathcal {T} (\mathcal {Z} ')$ is an artinian scheme (with support in the unique point of $\mathcal {N} _\textrm{red}$). Then, by [23, Cor. 5.5], we have

7.4.6 $$\begin{aligned} \langle [\mathcal {Z} ], \mathcal {T} _*([\mathcal {Z} '])\rangle _{\mathcal {N}}=\langle \mathcal {Z}, \mathcal {T} *\mathcal {Z} '\rangle _{Z^1(\mathcal {N})}, \end{aligned}$$

Here on the LHS appears the intersection product in K-theory (comp. (6.1.2)), and on the RHS appears the intersection number of properly intersecting cycles of codimension one on $\mathcal {N}$, cf. [23, Rem. 5.3].

We now return to the AFL. Recall that $\Delta \subset \mathcal {N} _1\times \mathcal {N} _2$ is the graph of the closed immersion $\mathcal {N} _1\hookrightarrow \mathcal {N} _2$. Under the identification $\mathcal {N} _1\times \mathcal {N} _2\simeq \mathcal {N}$, we can identify $\Delta$ with $\mathcal {Z} (u_0)$.

#### Corollary 7.4.3

Let $m\ge 1$. Then for all $g\in \textrm{U}(\mathbb {V} _2)$ regular semisimple,

$$\begin{aligned} {{\,\mathrm{\textrm{Int}}\,}}(g, \phi _m)=\langle g\Delta , (\mathcal {T} ^\circ _{m})_*(\Delta ) \rangle _{\mathcal {N}}=1. \end{aligned}$$

#### Proof

By $\Delta =\mathcal {Z} (u_0)$ and Proposition 7.4.2, we have by (7.4.6)

$$\begin{aligned} {{\,\mathrm{\textrm{Int}}\,}}(g, \phi _m)&=\langle g\Delta , \mathcal {T} ^\circ _{m}*\Delta \rangle _{Z^1(\mathcal {N})}=\langle \mathcal {Z} (g\cdot u_0), \mathcal {Z} (\varpi ^m u_0)^\circ \rangle _{Z^1(\mathcal {N})}\\&=\langle \mathcal {Z} (g\cdot u_0), \mathcal {Z} (\varpi ^m u_0) \rangle _{Z^1(\mathcal {N})}-\langle \mathcal {Z} (g\cdot u_0), \mathcal {Z} (\varpi ^{m-1} u_0) \rangle _{Z^1(\mathcal {N})}.\nonumber \end{aligned}$$

It remains to compute $\langle \mathcal {Z} (g\cdot u_0), \mathcal {Z} (\varpi ^m u_0)) \rangle _{Z^1(\mathcal {N})}$ for all $m\ge 0$. We can use [17]. The fundamental matrix of the lattice $\langle {g\cdot u_0, \varpi ^m u_0 } \rangle$ for the obvious basis is

$$\begin{aligned} A=\begin{pmatrix} 1 &  \varpi ^m(g\cdot u_0, u_0 ) \\ \varpi ^m( u_0,g\cdot u_0 )&  \varpi ^{2m} \end{pmatrix} \end{aligned}$$

and of the lattice $\langle {g\cdot u_0, \varpi ^{m-1} u_0 } \rangle$ is

$$\begin{aligned} A'=\begin{pmatrix} 1 &  \varpi ^{m-1}(g\cdot u_0, u_0 ) \\ \varpi ^{m-1}( u_0,g\cdot u_0 )&  \varpi ^{2(m-1)} \end{pmatrix}. \end{aligned}$$

Note that the valuation of $g\cdot u_0$ is zero and the space $\mathbb {V}$ is non-split (hence ${\textrm{val}}(\det A)$ is odd). It follows that $(g\cdot u_0, u_0 )$ is a unit, and the fundamental invariants of *A* (resp. $A'$) are $(0, {\textrm{val}}(\det A))$ (resp. $(0,{\textrm{val}}(\det A)-2)$). Then by [17] we obtain

$$\begin{aligned} \langle \mathcal {Z} (g\cdot u_0), \mathcal {Z} (\varpi ^m u_0)) \rangle _{Z^1(\mathcal {N})}=\frac{{\textrm{val}}(\det (A))+1}{2} \end{aligned}$$

and

$$\begin{aligned} \langle \mathcal {Z} (g\cdot u_0), \mathcal {Z} (\varpi ^{m-1} u_0)) \rangle _{Z^1(\mathcal {N})}=\frac{{\textrm{val}}(\det (A))-1}{2}. \end{aligned}$$

The assertion follows. $\square$

#### Remark 7.4.4

Note that $\mathbb {T} _{\le 2}$ is the composition of intertwining Hecke correspondences $\mathbb {T} _2^+$ and $\mathbb {T} _2^-$ which correspond to elements in the Iwahori Hecke algebra. One may try to generalize the result above from the spherical Hecke algebra to the Iwahori Hecke algebra; we have not done so.

### Comparison

#### Theorem 7.5.1

Conjecture 6.1.4 holds when $n=1$, namely AFL holds for the full Hecke algebra when $n=1$.

#### Proof

When $n=1$, the Hecke algebra $\mathcal {H} _{K^\flat }$ is trivial. Hence it suffices to show the inhomogeneous AFL conjecture 6.2.1. We show the identity for *f* running through the basis $\phi _m$ of $\mathcal {H} _K$.

By Lemma 7.1.1 (iii), we have $(\textrm{BC}^{\eta }_S)^{-1}(\phi _m)={\tilde{\varphi }}'_m\in \mathcal {H} _{K'_S}$ and hence we need to show that for matching *g* and $\gamma$,

$$\begin{aligned} {{\,\mathrm{\textrm{Int}}\,}}(g, \phi _m)\cdot \log q=-\omega (\gamma )\partial \!\operatorname {Orb}(\gamma ,{\tilde{\varphi }}'_{m}). \end{aligned}$$

When $m\ge 1$, this follows from directly comparing Proposition 7.3.2 (for orbital integrals) and Corollary 7.4.3 (for intersection numbers). More precisely, without loss of generality, we let $$\gamma = \begin{pmatrix} a &  b\\ c &  d \end{pmatrix}\in S_2(F_0)_\textrm{rs} $$ with $v(c)=0$. Then it matches an element in $U(\mathbb {V} _2)_\textrm{rs}$ if and only if $v(bc)=v(1-a\overline{a})$ is odd (in particular $v(a)\ge 0$). Recall from [31, Sect. 2.4] that the transfer factor is defined as

$$\begin{aligned} \omega (\gamma )=(-1)^{v(\det (e,\gamma e))}, \end{aligned}$$

where $e=(0,1)^t$ and hence $$\det (e,\gamma e)=\det \begin{pmatrix} 0 &  b\\ 1&  d \end{pmatrix}=-b$$. Therefore, when $v(b)=v(1-a{\bar{a}})$ is odd, the transfer factor is $\omega (\gamma )=(-1)^{v(b)}=-1$ and hence, by Proposition 7.3.2, (ii),

$$\begin{aligned} -\omega (\gamma )\partial \!\operatorname {Orb}(\gamma ,{\tilde{\varphi }}'_{m})=\log q. \end{aligned}$$

On the other hand, by Corollary 7.4.3 we have

$$\begin{aligned} {{\,\mathrm{\textrm{Int}}\,}}(g, \phi _m)=1 \end{aligned}$$

for all $g\in U(\mathbb {V} _2)_\textrm{rs}$, which proves the desired identity.

The case $m=0$ is the AFL (for the unit element in Hecke algebra) proved in [40]. We can also see this directly by using the facts proved in this section. The proof of Corollary 7.4.3 shows

$$\begin{aligned} {{\,\mathrm{\textrm{Int}}\,}}(g, \phi _0)=\langle g\Delta , \Delta \rangle _{Z^1(\mathcal {N})}&=\langle \mathcal {Z} (g\cdot u_0), \mathcal {Z} ( u_0) \rangle _{Z^1(\mathcal {N})} \\  &=\frac{{\textrm{val}}(\det (A))+1}{2} , \end{aligned}$$

where

$$\begin{aligned} A=\begin{pmatrix} 1 &  (g\cdot u_0, u_0 ) \\ ( u_0,g\cdot u_0 )&  1 \end{pmatrix}. \end{aligned}$$

Note that $v(\det (A))=v(1-a\overline{a})$, in terms of the matrix form $$g= \begin{pmatrix} a &  b\\ c &  d \end{pmatrix}\in U(\mathbb {V} _2)_\textrm{rs} $$ using the basis $\{u_0,u_1\}$.

On the other hand, by Proposition 7.3.2 (ii), for $$\gamma = \begin{pmatrix} a &  b\\ c &  d \end{pmatrix}\in S_2(F_0)_\textrm{rs} $$ we have

$$\begin{aligned} \partial \!\operatorname {Orb}(\gamma ,{\tilde{\varphi }}'_0)= \log q \left\{ \begin{array}{ll} \frac{v(1-a\overline{a})+1}{2}, &  v(1-a\overline{a})\ge 0\\ 0, &  v(1-a\overline{a})<0. \end{array}\right. \end{aligned}$$

Matching *g* and $\gamma$ have the same *a*. The computation of the transfer factor is the same as in the case $m\ge 1$. It follows that

$$\begin{aligned} {{\,\mathrm{\textrm{Int}}\,}}(g, \phi _0)\cdot \log q=-\omega (\gamma )\partial \!\operatorname {Orb}(\gamma ,{\tilde{\varphi }}'_{0}) = \frac{{\textrm{val}}(1-a\overline{a})+1}{2}. \end{aligned}$$

The proof is complete. $\square$

#### Remark 7.5.2

The RZ space $\mathcal {N} _2$ is isomorphic to the Lubin–Tate deformation space in height two, cf. Remark 5.5.6. It is therefore natural to expect that the AFL for the full Hecke algebra is related to the linear AFL for the Hecke algebra of $\textrm{GL}_2$ considered by Li [22]. We do not see an immediate passage between the two statements.

## Base-point freeness

In this section, we comment on how much information on an element of the Hecke algebra is contained in either side of the AFL, i.e., in the functionals on the Hecke algebra defined by the derivative of orbital integrals, resp. by the intersection numbers. It turns out that there is a surprising contrast between the functional defined by orbital integrals and that defined by the derivative of orbital integrals.

### Orbital integrals

For orbital integrals, there is the following fact.

#### Proposition 8.1.1

The map

$$\begin{aligned} {{\,\textrm{Orb}\,}}: \mathcal {H} _{K^{\prime \flat }\times K'}\rightarrow C^\infty (G'_{\textrm{rs}}) \end{aligned}$$

is injective.

#### Proof

We use a theorem of Beuzart-Plessis [1, Cor. 4.5.1]: *For any* $f'\in C_c^\infty (G')$, *the orbital integral* ${{\,\textrm{Orb}\,}}(-,f')$ *vanishes at all regular semisimple elements if and only if* $I_{\Pi }(f')=0$ *for all* $\Pi \in \Phi _{\textrm{temp}}(G'(F_0))$. Here $\Phi _{\textrm{temp}}(G'(F_0))$ denotes the set of irreducible tempered admissible representations of $G'(F_0)$, and $I_{\Pi }(f')$ is the local relative character associated to the local Rankin–Selberg period integral and the local Flicker–Rallis period integral.

Let $f'\in \mathcal {H} _{K^{\prime \flat }\times K'}$ with identically vanishing orbital integrals. Then by the above quoted theorem, $I_{\Pi }(f')=0$ for all $\Pi$ as above. In particular, $I_{\Pi }(f')=0$ for all $\Pi \in \Phi ^{\textrm{ur}}_{\textrm{temp}}(G'(F_0))$, the set of tempered unramified representations. However, for $\Pi \in \Phi ^{\textrm{ur}}_{\textrm{temp}}(G'(F_0))$, we have $I_{\Pi }(f')=\lambda _\Pi (f') I_\Pi (\textbf{1}_{{K'}})$. Here $\lambda _\Pi : \mathcal {H} _{K^{\prime \flat }\times K'}\rightarrow \mathbb {C}$ is the algebra homomorphism associated to $\Pi$ or, equivalently, the evaluation of $\textrm{Sat}(f')$ at the Satake parameter of $\Pi$. Note that $I_\Pi (\textbf{1}_{{K'}})\ne 0$ for $\Pi \in \Phi ^{\textrm{ur}}_{\textrm{temp}}(G'(F_0))$. Hence $\lambda _\Pi (f')=0$ for all $\Pi \in \Phi ^{\textrm{ur}}_{\textrm{temp}}(G'(F_0))$. It follows that $f'=0$ by the Satake isomorphism. $\square$

### Derivative of orbital integrals

Let $G'_{\textrm{rs}, W_1}$ denote the open subset of $G'_{\textrm{rs}}$ consisting of regular semisimple elements matching with elements in the non-quasi-split unitary group $G_{W_1}$.

#### Conjecture 8.2.1

The map

$$\begin{aligned} \partial \!\operatorname {Orb}: \mathcal {H} _{K^{\prime \flat }\times K'}\rightarrow C^\infty (G'_{\textrm{rs}, W_1}) \end{aligned}$$

has a large kernel, in the sense that the kernel generates the whole ring $\mathcal {H} _{K'}$ as an ideal (note that this kernel is only a vector subspace rather than an ideal). Similarly, the map defined by the intersection numbers, ${{\,\mathrm{\textrm{Int}}\,}}: \mathcal {H} _{K^\flat \times K}\rightarrow C^\infty (G'_{\textrm{rs},W_1})$, has a large kernel.

#### Remark 8.2.2

A weaker conjecture would be that “the kernel is not contained in any maximal ideal of $\mathcal {H}$ corresponding to a tempered representation". Here a $\mathbb {C}$-point $\alpha$ of ${{\,\textrm{Spec}\,}}\mathcal {H} _{K'}$ is called tempered if, in terms of the coordinates in Sect. 3.4, we have $|\alpha _i|=1$ for all *i*. There might be some other ways to formulate the smallness of the image. On the other hand, the image should not be too small, although we do not have a precise conjecture. In general it is unclear to us how to characterize the image.

#### Theorem 8.2.3

Conjecture 8.2.1 holds when $n=1$.

#### Proof

When $n=1$, the factors $\mathcal {H} _{K'^\flat }$ and $\mathcal {H} _{K^\flat }$ are trivial. We note that the orbital integral map $\partial \!\operatorname {Orb}$ factors through the base change homomorphism $\textrm{BC}$. We now identify the set of orbits in $G'_{\textrm{rs}}$ with the set of orbits in $S_{\textrm{rs}}$. The induced map

8.2.1 $$\begin{aligned} \partial \!\operatorname {Orb}_G: \mathcal {H} _{K}\rightarrow C^\infty (G'_{\textrm{rs}, W_1}) \end{aligned}$$

can be written in terms of

$$\begin{aligned} \partial \!\operatorname {Orb}_G(-,\phi ):=\omega _{S}(-)\partial \!\operatorname {Orb}(-,(\textrm{BC}^{\eta }_S)^{-1}(\phi )),\quad \phi \in \mathcal {H} _{K}. \end{aligned}$$

Then the assertion is equivalent to the statement that the kernel of $\partial \!\operatorname {Orb}_G$ generates the whole ring $\mathcal {H} _{K}$ as an ideal. We use the explicit results in Proposition 7.3.2, which shows that the image is exactly two dimensional, spanned by the image of $\phi _0$ and any one of the $\phi _m,\, m\ge 1$. In particular, the kernel of $\partial \!\operatorname {Orb}_G$ is spanned by the set $\{\phi _m-\phi _1\mid m\ge 2\}$. Equivalently it remains to show that the elements of this set have no common zero. From (7.1.4), we have for $m\ge 2$

$$\begin{aligned} \textrm{Sat}( \phi _m-\phi _1)=q^{m}\sum _{i=-m}^m X^{i}-q^{m-1}\sum _{i=-(m-1)}^{m-1} X^{i}- q(X+1+X^{-1})+1. \end{aligned}$$

It is straightforward to check that these Laurent polynomials have no common zero in $X\in \mathbb {C} ^\times$. Indeed, already these Laurent polynomials for $m=2$ and $m=3$ have no common zero. To see this, we can write these Laurent polynomials as polynomials $P_2$, resp. $P_3$, of degree 2, resp. 3, in $\mathbb {C} [X_1]$, where $X_1=X+X^{-1}$. Long division shows that these polynomials are coprime, hence there are polynomials $R_2, R_3\in \mathbb {C} [X_1]$ with $P_2R_2+P_3 R_3=1$. Rewriting this identity in terms of $X^{\pm 1}$ shows that the Laurent polynomials for $m=2, 3$ have no common zero. The proof is complete. $\square$

#### Remark 8.2.4

A similar result in the setting of the linear AFL (for $\textrm{GL}_2$ rather than $\textrm{U}_2$) could be deduced from the result of Q. Li [22, Prop. 7.6].

## Data Availability

Data sharing not applicable to this article as no datasets were generated or analysed during the current study.

## Appendix: Correspondences for formal schemes and maps on K-groups

In this appendix, we explain the calculus of correspondences that we use, comp. also [42, App. B]. Let $\breve{O}$ be a strictly henselian DVR. We consider locally noetherian formal schemes, locally formally of finite type over $\breve{O}$. For such a formal scheme $\mathcal {X}$ and a closed formal subscheme *A* of $\mathcal {X}$, we denote by $K^A(\mathcal {X})$ the Grothendieck group of finite complexes of locally free modules over the structure sheaf which are formally acyclic outside *A*, comp. [11]. Recall from [41, App. B] that this means that the cohomology sheaves of *C* are annihilated by a power of the ideal sheaf of *A*, locally on $\mathcal {X}$. We similarly have the Grothendieck group $K'(A)$ formed by finite complexes of coherent modules on *A*.

### Induced map on K-groups

Let $\mathcal {X}$ and $\mathcal {Y}$ be formal $\breve{O}$-schemes as above. Let $\mathcal {T} \rightarrow \mathcal {X} \times _{\breve{O}} \mathcal {Y}$ be a *geometric correspondence*, i.e., a formal scheme as specified above, with a morphism of formal schemes as indicated. We also simply write $\mathcal {T}$ for this correspondence and denote by $p_\mathcal {X}$, resp. $p_\mathcal {Y}$, the maps to $\mathcal {X}$, resp. $\mathcal {Y}$. We assume that $\mathcal {Y}$ is regular and that $p_\mathcal {Y}$ is a proper morphism. Then $\mathcal {T}$ defines a map on K-groups with supports,

9.1.1 $$\begin{aligned} \mathcal {T} _*:K^A(\mathcal {X})\rightarrow K^{\mathcal {T} (A)}(\mathcal {Y}), \quad x\mapsto Rp_{\mathcal {Y}, *}(p_\mathcal {X} ^*(x)). \end{aligned}$$

Here $\mathcal {T} (A)=p_\mathcal {Y} (p_\mathcal {X} ^{-1}(A))$, i.e., $\mathcal {T} (A)$ is the image of the closed formal subscheme $p_\mathcal {X} ^{-1}(A)$ of $\mathcal {T}$ under the proper morphism $p_\mathcal {Y}$. This image is by definition the closed formal subscheme of $\mathcal {Y}$ defined by the kernel ideal of the map $\mathcal {O} _\mathcal {Y} \rightarrow p_{\mathcal {Y}, *}(\mathcal {O} _\mathcal {T})$. Note here that by the properness of $p_\mathcal {Y}$, the target of this map is a coherent $\mathcal {O} _\mathcal {Y}$-module. We note that $K^A(\mathcal {X})$ only depends on the radical of the ideal sheaf defining the closed formal subscheme *A*. Let us explain the construction of the map. The map is the composition of the maps

9.1.2 $$\begin{aligned} p_\mathcal {X} ^*:K^A(\mathcal {X})\rightarrow K^{p_\mathcal {X} ^{-1}(A)}(\mathcal {T}), \quad p_{\mathcal {Y}, *}:K^{p_\mathcal {X} ^{-1}(A)}(\mathcal {T})\rightarrow K^{\mathcal {T} (A)}(\mathcal {Y}). \end{aligned}$$

For the first map, let $x\in K^A(\mathcal {X})$ be represented by a finite complex *C* of locally free $\mathcal {O} _\mathcal {X}$-modules formally acyclic outside *A*. Then its base change to $\mathcal {T}$ is a finite complex of locally free $\mathcal {O} _\mathcal {T}$-modules which is formally acyclic outside $p_\mathcal {X} ^{-1}(A)$, cf. [11, Sect. 1.5]. The image $p_\mathcal {X} ^*(x)$ is defined to be the class of this complex. The second map is the composition of three maps. First, the natural map $K^{p_\mathcal {X} ^{-1}(A)}(\mathcal {T})\rightarrow K'(p_\mathcal {X} ^{-1}(A))$, sending a complex *C* which is formally acyclic outside a closed subset to the alternating sum of the classes in $K'$ of the cohomology sheaves of *C*. Second, the full direct image map $K'(p_\mathcal {X} ^{-1}(A))\rightarrow K'(p_\mathcal {Y} (\mathcal {T} (A)))$, defined by the properness of $p_\mathcal {Y}$. Third, the identification $K'(\mathcal {T} (A))=K^{\mathcal {T} (A)}(\mathcal {Y})$ by the regularity of $\mathcal {Y}$, cf. [11, Lem. 1.9].

### Composition

Recall the composition of geometric correspondences. Let $\mathcal {T} \rightarrow \mathcal {X} \times _{\breve{O}} \mathcal {Y}$ and $\mathcal {S} \rightarrow \mathcal {Y} \times _{\breve{O}} \mathcal {Z}$ be geometric correspondences as above. The composition $\mathcal {U} =\mathcal {S} \circ \mathcal {T}$ of these correspondences $\mathcal {T}$ and $\mathcal {S}$ is defined by the following diagram with cartesian square,

In other words, the projections for $\mathcal {U}$ are $r_\mathcal {X} =p_\mathcal {X} \circ q'_\mathcal {Y}$ and $r_\mathcal {Z} =q_\mathcal {Z} \circ p'_\mathcal {Y}$.

Assume now that $\mathcal {Y}$ and $\mathcal {Z}$ are regular and the morphisms $p_\mathcal {Y}$ and $q_\mathcal {Z}$ proper, so that also $r_\mathcal {Z}$ is proper. Let *A* be a closed formal subscheme of $\mathcal {X}$. Then the three maps are defined,

$$\begin{aligned}  &   \mathcal {T} ^A_*:K^A(\mathcal {X})\rightarrow K^{\mathcal {T} (A)}(\mathcal {Y}), \quad \mathcal {S} ^{\mathcal {T} (A)}_*:K^{\mathcal {T} (A)}(\mathcal {Y})\rightarrow K^{\mathcal {U} (A)}(\mathcal {Z}), \quad \\  &   \quad \mathcal {U} ^A_*:K^A(\mathcal {X})\rightarrow K^{\mathcal {U} (A)}(\mathcal {Z}). \end{aligned}$$

#### Lemma 9.2.1

Assume that the maps $p_\mathcal {Y}$ and $q_\mathcal {Y}$ are tor-independent, i.e., $\mathcal {Tor}_j^{\mathcal {O} _\mathcal {Y}}(\mathcal {O} _\mathcal {T}, \mathcal {O} _\mathcal {S})=0, \forall j>0$, cf. [33, Def. 36.22.2]. Then

$$\begin{aligned}\mathcal {U} ^A_*=\mathcal {S} ^{\mathcal {T} (A)}_*\circ \mathcal {T} ^A_*:K^A(X)\rightarrow K^{\mathcal {U} (A)}(Z). \end{aligned}$$

#### Proof

We need to show that

$$\begin{aligned} Rq_{\mathcal {Z}, *}\big (Rp'_{\mathcal {Y}, *}({q'}_\mathcal {X} ^*(p_\mathcal {X} ^*(x)))\big )=Rq_{\mathcal {Z}, *}\big (q_\mathcal {Y} ^*(Rp_{\mathcal {Y}, *}(p_\mathcal {X} ^*(x)))\big ). \end{aligned}$$

Here $q_\mathcal {Y} ^*(Rp_{\mathcal {Y}, *}(p_\mathcal {X} ^*(x)))$ makes sense as an element in $K(\mathcal {S})$ since, by the regularity of $\mathcal {Y}$, the complex $Rp_{\mathcal {Y}, *}(p_\mathcal {X} ^*(x))$ can be interpreted as an element in $K(\mathcal {Y})$. It therefore suffices to prove the equality of elements in $K'(\mathcal {S})$,

$$\begin{aligned} Rp'_{\mathcal {Y}, *}\big ({q'}_\mathcal {X} ^*(p_\mathcal {X} ^*(x))\big )=q_\mathcal {Y} ^*\big (Rp_{\mathcal {Y}, *}(p_\mathcal {X} ^*(x))\big ). \end{aligned}$$

This follows from base change for the cartesian square, again representing $Rp_{\mathcal {Y}, *}(p_\mathcal {X} ^*(x))$ by a finite complex of locally free $\mathcal {O} _\mathcal {Y}$-modules, cf. [33, Lem. 36.22.5]. $\square$

#### Remark 9.2.2

We use this lemma only in the case when one of the two morphisms $p_\mathcal {Y}$ and $q_\mathcal {Y}$ is flat. In general, the identity $\mathcal {U} ^A_*=\mathcal {S} ^{\mathcal {T} (A)}_*\circ \mathcal {T} ^A_*$ does not hold. However, it does always hold in the context of *derived formal schemes*. Indeed, in this context, the base change formula used above always holds, comp. [7, part III, ch. 3, Prop. 2.2.2].

## Footnotes

1 The terminology “intertwining Hecke function" is borrowed from [24, Definition B.2.3] where a special case is considered.

2 It may be possible, at least as far as defining and calculating intersection multiplicities is concerned, to replace derived formal schemes by their underlying classical formal scheme, equipped with a suitable element in the derived category of coherent sheaves.

3 Note that in [20], the twist $\eta ^{n}$ is erroneously omitted.
